# Functional diversity exhibits a diverse relationship with area, even a decreasing one

**DOI:** 10.1038/srep35420

**Published:** 2016-10-18

**Authors:** Elpida K. Karadimou, Athanasios S. Kallimanis, Ioannis Tsiripidis, Panayotis Dimopoulos

**Affiliations:** 1 Department of Environmental and Natural Resources Management, University of Patras, Seferi 2, GR-30100, Agrinio, Greece; 2 School of Biology, Aristotle University of Thessaloniki, GR-54124, Thessaloniki, Greece

## Abstract

The relationship between species richness and area is one of the few well-established laws in ecology, and one might expect a similar relationship with functional diversity (FD). However, only a few studies investigate the relationship between trait-based FD and area, the Functional Diversity - Area Relationship (FDAR). To examine FDAR, we constructed the species accumulation curve and the corresponding FD curve. We used plant diversity data from nested plots (1–128 m^2^), recorded on the Volcanic islands of Santorini Archipelagos, Greece. Six multidimensional FD indices were calculated using 26 traits. We identified a typology of FDARs depending on the facet of FD analyzed: (A) strongly positive for indices quantifying the range of functional traits in the community, (B) negative correlation for indices quantifying the evenness in the distribution of abundance in the trait space, (C) no clear pattern for indices reflecting the functional similarity of species and (D) idiosyncratic patterns with area for functional divergence. As area increases, the range of traits observed in the community increases, but the abundance of traits does not increase proportionally and some traits become dominant, implying a reliance on some functions that may be located in either the center or the periphery of the trait space.

Recently, biodiversity research has focused on more integrative approaches that take into account not only species richness, but also species traits in an attempt to quantify the community’s functional diversity^1^,^2^,^3^,^4^. Functional diversity indices quantify the trait diversity and act as a surrogate for the diverse ecological functions performed in the community. The diversity of trait values within a community is therefore referred as either trait diversity or functional diversity (FD). Here we use the latter term for consistency with most of the literature studying ecosystem functioning^5^ and community-level assembly processes^3^,^6^. Functional diversity is a multifaceted concept that includes many different phenomena. The most widely used aspect of FD reflects the range of the traits of the organisms in a community, i.e. the amount of trait space that the species in a given community occupy. Τhis facet aims to estimate the amount of niche space filled by the community and it is measured by the indices Functional Richness and dendrogram based FD^7^. But this aspect of FD does not take into account the species abundance distribution and gives equal weight to rare and common species, even though the latter are expected to play a more significant role in ecosystem functioning. Thus other facets of FD focus on the distribution of individuals in this trait space. Functional evenness (FEve) reflects how evenly individuals are distributed in the trait space, i.e. if all the trait space is equally represented^7^, or if some traits are overrepresented while others are underrepresented. Another important aspect of FD is the degree to which abundance distribution in trait space maximises towards the extremes or the center of this occupied trait space^3^. One typical index of this facet is Functional divergence (FDiv). Finally another facet of FD reflects the similarity (in trait space) among the species in the community. This may take into consideration the species abundances. This aspect of FD is based on the degree of functional dissimilarity between members of the community and is measured using Rao’s quadratic entropy (RaoQ)^8^ and functional dispersion (FDis)^9^.

Functional diversity, measured in terms of volume of the functional trait space occupied by the species assemblage, is strongly related to species richness^10^,^11^,^12^,^13^. In contrast, other aspects of FD (especially those taking into account the species abundance distribution) are often weakly or not significantly correlated to species richness^10^,^11^,^12^. In general, we still have limited knowledge on the patterns and drivers of FD. For example, it is still debatable if well documented patterns of species richness (such as the species - area relationship) apply to FD indices and to what extent, and if there are deviations, when do they occur and which are the underlying mechanisms driving them.

The intuitive assumption is that the Functional Diversity - Area Relationship (FDAR) is similar to the species - area relationship (SAR). To date, there are only few examples documenting a strong positive correlation between FD and area, a relationship that resembles the SAR pattern^13^,^14^,^15^,^16^. The FD indices used in these studies reflect the volume of functional space occupied by the community, i.e. the facet of functional diversity that is strongly correlated to species richness. Whang, *et al*.^14^ suggest that a more comprehensive effort to investigate FDAR should incorporate abundance-weighted multidimensional metrics of FD. In a first effort to use abundance-weighted indices in the FDAR analysis for the identification of global hotspots, Mazel, *et al*.^15^ show that, when incorporating relative species coverage, species richness and FD display differing patterns and therefore are not good surrogates of each other. All these examples refer to comparisons among independent sites of different areas (island type SAR). To date, there are no studies investigating the type of correlation between the species accumulation curves and the FD accumulation curves.

The increase of diversity with increasing area is one of the few general laws in ecology^17^,^18^. Based on this knowledge, it could be assumed that as area increases functional diversity should also increase, especially in the case of nested plots, where species accumulate. But given that different facets of FD display different relationships with species richness, we hypothesize that different facets of FD display different patterns of FDAR. Functional diversity measured in terms of the volume of functional trait space is strongly related to species richness and thus we hypothesize that the FDARs constructed using these metrics strongly resemble the well-known SAR. Furthermore, we hypothesize that abundance-weighted FD measures display considerably more diverse FDAR patterns. On the one hand, as area increases a larger number of rare species will be discovered, and the common species will become more dominant thus skewing the species abundance distribution, and decreasing the evenness facet of FD. But on the other hand, theoretically it is also possible that as area increases, new habitats will be found, and along with them new common species. Thus the formerly common species will become less dominant while the formerly rare species may become more common resulting in a decrease of the skeweness of the species abundance distribution, and an increase of the evenness facet of FD. Also, the intuitive assumption would be that the common species are the ones that are best adapted to the area’s conditions, while the other species in the community are slightly less well adapted and thus, in the trait space, they are located around the space occupied by the common species. In other words, we expect that the common species will be located in the center of the community’s trait space, and therefore as area increases and more species of the community are examined functional divergence will increase.

In this study, we examine the relationship between functional diversity and area (FDAR). More specifically, we investigate how the different facets of FD change with increasing area. We test the hypothesis that this relationship reflects and resembles the corresponding species - area relationship. To this end we used nested plots to construct the functional diversity accumulation curves at fine scales (1–128 m^2^). Then we compared this relationship with the species accumulation curves or type I SAR according to Scheiner^19^. These curves were constructed using abundance data from 16 macroplots, each consisting of 17 subplots of different scales. For each scale and plot we measured not only species richness, but also quantified and estimated various facets of FD, using six indices that measure the diversity and evenness of the trait space occupied by the species in the community^20^,^21^. In our study we used 26 traits to compute FD (for details on the traits used see Supplementary Table S1).

## Results

### The aspect of FD analyzed affects the pattern of nested FDAR

Spearman rank correlations among the values of the six indices indicate that most of the indices’ values are highly correlated (see Supplementary Table S3). All 26 traits are well represented by the four axes of the PCoA analysis that were used as the new synthetic traits for the computation of FD indices. The 1^st^ axis represents 28.92% of the variation of traits, the 2^nd^ 24.45%, the 3^rd^ 20.54% and the 4^th^ 16.69%.

**Figure 1:**
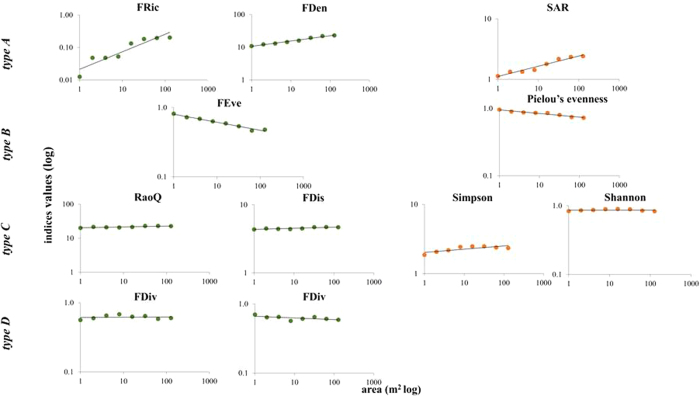
The relationship between biodiversity and area (1–128 m^2^), using accumulation curves for nested plots. Biodiversity is estimated using both trait-based functional diversity indices (Green dots) and taxonomic diversity indices (Orange dots). Power model fit is given for each curve. The plots’ axes are represented in a log-log scale. Accumulation the curves for one of the 16 plots are presented here; the curves for the remaining plots follow the same pattern and could be found in supplementary material. Each row corresponds to one of the four types of pattern detected for this relationship: **Type A** is represented by functional diversity indices FRic (functional richness) and FDen (functional dendrogram) which display a positive trend with area. This pattern strongly resembles the pattern of the species – area relationship. The same positive trend is detected across the plots of all four communities. **Type B** is represented by the functional diversity index FEve (functional evenness) which display negative correlation with area, regardless of the plot or the community examined. This pattern strongly resembles the one displayed by Pileou’s evenness diversity index. **Type C** is represented by functional diversity indices RaoQ (Rao’s quadratic entropy) and FDis (functional dispersion) which (depending on the plot) display weak to no significant correlation with area. Similar is the pattern displayed by the Simpson and Shannon diversity indices. **Type D** is represented by the index FDiv (functional divergence) which display negative, positive or no correlation with area, depending on the plot and community examined.

Slope values for the FRic and FDen indices’ relationship with area are comparable to those of the SARs, although FDARs are less steep (Table 1). The relationship between the various FD indices and area can be classified into four types. Type A consists of FRic and FDen indices, which display strong positive correlation with area, similar to the well-known pattern of SAR (here displayed using the species – accumulation curve) (Fig. 1). The other types include the indices that take into account species abundances. The latter indices display FDAR patterns that do not resemble the SAR patterns (Fig. 1). In particular, type B is represented by the index FEve, which in most cases displays statistically significant negative correlations with area (Fig. 1). Type C, which includes FDis and RaoQ indices, displays FDAR patterns that resemble the relationship between area and the Shannon or Simpson diversity index (Fig. 1). Finally, type D (FDiv index) displays weak or no correlation with area (Fig. 1). The functional diversity accumulation curves of all 16 plots are presented graphically in the Supplementary material (Figs S1, S2, S3 and S4).

In type A FDAR (FRic, FDen), FD increases as area increases (Fig. 1). The relationship is best fitted by the power law model (Table 2). At the scales of our analysis, there is no evidence of saturation in the FDAR pattern. This pattern strongly resembles the species – accumulation curve (Fig. 1). Thus, it is not surprising that these FD indices also display a strong positive relationship with species richness. We used the power model to compare the slopes (z values) of the lines described by the equation, since this model is the best fit in the majority of plots (Table 2, see also Supplementary Table S2). The species – accumulation curve lines display z values ranging between 0.11–0.51, while the corresponding values for FRic are higher, ranging between 0.63 and 1.63, and for FDen lower, ranging between 0.08–0.34.

The type B FDAR - a consistently negative correlation with area - includes FEve index, which is the only index systematically displaying this negative relationship with area (Fig. 1, second row). This FDAR pattern resembles in many plots the relationship between Pielou’s evenness index and area. In this type, there is no best fitted model, since in different plots each of the power, polynomial and logarithmic models provided best fit, with R^2^ values ranging between 0.51 and 0.97 (see Supplementary Table S2). FEve is also significantly negatively correlated with species richness.

The type C FDAR is the case of FDis and RaoQ indices which behave similarly and are highly correlated because they are computed in a corresponding way. In some plots these indices do not display significant correlation with area, although in a few plots there is a positive correlation with area and species richness (Fig. 1, third row). In some cases, their values initially decrease with increasing area and then increase at coarser scales. Their FDAR pattern resembles in most cases the relationship between area and the Simpson or Shannon diversity indices (Fig. 1). Linear, polynomial and power models best fitted the FDARs of the FDis and RaoQ indices, with R^2^ > 0.9 in the majority of plots (see Supplementary Table S2).

Finally, the type D FDAR (FDiv index) displays a more idiosyncratic response to area. FDiv displays various patterns that can be ascending, descending or independent of area (Fig. 1). In this case, R^2^ is low (<0.5, in the majority of plots) since, for most of the models examined, FDiv values are not significantly correlated with area (see Supplementary Table S2).

Trait selection is a critical step in FD studies affecting the patterns of FD observed^22^. Therefore we divided our traits dataset into three subsets: (i) vegetative characteristics, (ii) ecological preferences (based on Ellenberg indices), and (iii) regenerative characteristics (Supplementary Table S1), and then compared the FDAR patterns of these subsets with the FDAR patterns of the entire dataset. For type A FDAR, trait selection does not appear to play an important role, and the pattern is not affected (Supplementary Figure S5). For type B and C FDARs, the main patterns remain unaffected, even though there are few examples where one set of traits produce different FDAR compared to the other sets. These deviations are observed only at the finest scales with the lowest species richness values (Supplementary Figure S5). The type D FDAR displays divergent patterns depending on the traits used in the analysis (Supplementary Figure S5). For the same plot, the FDAR is increasing or decreasing depending on the traits used.

## Discussion

Functional richness increases with area. The indices that account for the range of functional traits within the assemblage, without being weighted by abundance, reflect that as area increases, so does the range of trait values and the trait differences between the species in the community. But if we take into account the species abundance distribution, diverse behaviors between FD and area are observed. We distinguish four types of functional diversity – area relationships for nested plots at small spatial scales. The scale of our analysis implies that these patterns refer mainly to species occupancy and not species turnover^23^.

As area increases, the amount of available energy increases, and thus the amount of resources available to the species assemblage increases as well, leading to more species-rich communities^24^. As species accumulate the volume of trait space occupied by the community remains either constant or increases, leading, in the second case, to a strong positive relationship between FD (quantified as FRic or FDen, i.e. indices that reflect the range of functional traits recorded in a community without taking into account the relative abundance of species) and area. These increased FD values may imply that more niches become available at coarser scales (perhaps as a result of greater energy availability or greater environmental heterogeneity). The increased FD implies that the community utilizes the available resources more efficiently, thus is better buffered against environmental fluctuations or against establishment of invasive species^25^.

Type A FDAR is typical of the most widely used indices of functional diversity (FRic and FDen). The relationship between species richness and FRic and FDen has been often documented^7^,^10^,^11^,^26^,^27^,^28^,^29^. Our results are in accordance with these findings, since FRic and FDen increase almost linearly with species richness and there is no evidence of saturation in this relationship, for the majority of plots. In our analysis, these patterns appear to be independent of the traits used to quantify FD. This type of FDAR has already been documented in FDAR studies that quantified FD either as FRic or as FDen using independent areas and non-nested plots (i.e. island type SARs)^13^,^14^,^15^,^16^. The slope of the FDARs, as far as FRic and FDen are concerned, is comparable to the SARs, although it is less steep. The SARs z-values fluctuate close to the spectrum of values reported in the literature^30^. Mazel, *et al*.^15^, studying the FDAR among ecoregions (at coarser scales), report that FD generally reaches its maximum value faster than species richness as a function of area, arguing that FD might be less vulnerable to habitat loss than species richness. Our results show a different trend, i.e. for the small scales used in our analysis, both species richness and FRic or FDen seem to reach their maximum values, but no saturation, at the same scales and thus they seem to be equally vulnerable to habitat loss.

The most counterintuitive type of FDAR is type B, where FD decreases as area increases. This type is observed in the case of FEve index that reflects the evenness of the distribution among individuals in the functional trait space and takes into account species abundances. As scale increases, more rare species are recorded (increasing the total species richness and the functional trait space occupied). Simultaneously, more individuals of the common species are recorded thus the species abundance distribution at coarser scales is more skewed. This means that parts of the functional trait space are empty while others are overrepresented. Macroecology reports the omnipresence of some statistical regularities across taxa, scales and biogeographical regions^31^. Among them, almost universal is the pattern that species abundance distributions are rightly skewed with many more rare species than common ones^31^,^32^. For FD, this pattern results in some traits being overrepresented in the community, while others virtually absent, and thus large parts of the trait space are left empty. At the fine scales of our study, as area increases the skewness in the distribution increases as well as the trait space occupied. This invariably leads to decreased evenness in the occupation of the trait space. The implication of this trend, for the scale of our analysis, is that as area increases, and despite the possible utilization of a wider range of resources, some resources may be underutilized while others overutilized, probably reflecting the availability of the different resources.

Type B FDAR is observed in the case of the FEve index that reflects the evenness of the distribution among individuals in the functional trait space and takes into account species abundances. FEve can therefore be compared to species evenness (Pielou’s J). This pattern does not appear to be dependent on trait selection, since, using different sets of traits, congruent patterns in almost all cases were produced, with the exception of plots with low species richness. This pattern may be relevant only to the scale of our analysis and it remains unknown if it would be observed at coarser scales, where an increase in area is associated with an increase in habitat diversity. This association between area and habitat diversity may result in a switch of dominance patterns and thus may result in less skewed species abundance distributions among habitats than within habitats. For example, bird functional evenness increases with landscape diversity^33^ and zooplankton functional evenness increases with habitat heterogeneity^34^. On the other hand, the colonization of new species in grasslands led to a decline in FEve through time^16^. Moreover, Ding, *et al*.^25^ analyzing the avifauna of islands of different size also report a negative FDAR pattern. Thus the generality of this pattern remains an open research question. A negative correlation has also been detected between species richness and species evenness at very small scales, but this negative relationship vanishes at larger scales^35^. Several studies show that scale can influence the measurement of species diversity and may alter the observed relationship between species richness and evenness^35^,^36^,^37^.

An important issue in biodiversity research is functional redundancy. The insurance hypothesis posits that many species in a community are similar and could potentially fill in the same functional role, and thus, if one species disappears there are other species tο fill the gap^38^. FDis and RaoQ are FD indices quantifying how functionally similar are the individuals in the trait space^39^. Their variation with scale indicates whether the new individuals added to the community are more or less similar to the existing ones. If redundancy is high, then the value of these indices is expected to remain constant or even decrease. However, if redundancy is low, the new individuals are less similar and the indices’ values should increase. In our case, the results cannot lead us to a firm conclusion, since for different plots we obtain different patterns. Moreover, new individuals may be similar regarding some traits but dissimilar regarding others, compared to the already existing individuals, and this might explain the differences that were observed when different traits were analyzed. Type C FDAR is characterized by contrasting patterns in our empirical data. In some cases there is a negative trend, similar to type B FDAR, indicating that at coarser scales species are more similar to the existing species at the finer scales; but in other cases a sigmoid trend is detected implying that initially new dissimilar species are added but after a point the newcomers are similar to the species already present. This type of FDAR closely resembles the relationship between diversity indices like Shannon or Simpson and area.

The last type of FDARs (showing weak negative or positive, or not significant correlations) was observed for FDiv that reflects the divergence of individuals in the functional space occupied by the species assemblage. Type D FDAR appears very idiosyncratic even among plots from the same community, displaying either weak positive or weak negative trends. Our results show that (within a plant community) the dominant species and their abundance do not differ significantly among the plots, but despite this, the FDAR pattern of FDiv does differ. The differences in the FDAR pattern among the plots examined is the result of the position of the common species in the functional trait space occupied by the entire species assemblage. If the dominant species are located towards the center of the functional space, occupied by the given species assemblage, then FDiv increases with area. But, if the dominant species are located towards the margins of the functional trait space the opposite trend is observed (FDiv decreases with area). This behavior underscores the role of the rare species of the community, since different plots with the same common species display different behavior. This finding highlights the importance of the community context for FDiv and thus it may explain why in most cases FDiv displays no clear patterns in contrast to other aspects of diversity^25^. The importance of the location of the dominant species in the functional trait space for the FDAR pattern of FDiv explains why this index is so sensitive to trait selection. For any given assemblage, different traits mean different trait space and this difference may affect the location of the dominant species in relation to the other species in the community. Thus, different traits lead to positive or negative FDAR for the same community even in the same nested plots.

Virtually everything important in biodiversity is tied up in issues of scale^23^,^24^. This is certainly true for species richness, where SAR is one of the few well established laws in ecology. This is also true for FD, but with the relationship being complex and dependent on the facet of FD analyzed. Before any generalizations are established, additional analysis should be made, examining more taxa, at different spatial scales and using different traits. Here, we analyzed how the different facets of FD change as area increases and species accumulate. As species accumulate, the volume of functional space occupied by the community either remains constant or increases, leading to FDAR patterns which are similar to SAR. But this space is not evenly occupied. As a matter of fact, as area increases more rare species are observed, while the abundance of common species increases disproportionately thus producing more right skewed species abundance distributions and thus decreasing functional evenness. As species accumulate, the newcomers are more similar (in the trait space) to the existing species, at least beyond a certain scale (since at coarse scales, the value of functional dispersion or Rao’s entropy does not increase), thus indicating the existence of a certain degree of functional redundancy among the species in the assemblage at coarser scales. As species accumulate, some traits become dominant, implying a reliance on some functions that may be located in either the center or the periphery of the trait space and thus producing idiosyncratic patterns in the relationship between functional divergence and area. As a general conclusion, the relationship between FD and area is complex and extrapolations should be done with caution.

## Methods

### Study area

The Santorini Archipelago (36°24′N and 25°24′E) is an active volcano of the South Aegean volcanic arc. The intracaldera volcanism of the last 2200 ys has formed the two Kameni Islands^40^: Palea Kameni (PK) that emerged in 46–47 A.D. and covers an area of 0.54 km^2^; and Nea Kameni (NK) that achieved its present shape (with an area of 3.44 km^2^) through eight eruptive phases between ca. 1570 and 1950 A.D. It is 1500 yr younger than PK. (for more details see Karadimou, *et al*.^6^ and Dimopoulos, *et al*.^41^).

Dimopoulos, *et al*.^41^ identified seven plant communities on both islands but only four out of the seven plant communities (the most common ones) were included in this study: three therophytic (*Lupinus angustifolius – Hyparrhenia hirta*, *Lupinus angustifolius – Helichrysum italicum* on ΝΚ and *Lupinus angustifolius – Tolpis barbata* on PΚ) and one community on PK that is dominated by the woody taxon *Pistacia lentiscus*, as they were the most extended and easier to reach. The two communities studied on NK cover approximately 20–30% of the island’s surface, while on PK 70–80%.

### Sampling

Multi-scale vegetation sampling was conducted on April 2010 using a set of 16 macroplots, comprised of 17 subplots of different size, four in each community (for more details on the sampling method and sampling plot figure see Karadimou, *et al*.^6^). To construct the species accumulation plot, we merged the original nearby subplots (of 1 m^2^, 4 m^2^, 16 m^2^ and 64 m^2^) of each macroplot of the same area successively, summing up their area and total species richness, starting from the smaller subplots using all possible combinations. This procedure resulted in 8 sub-plots with size 1 m^2^, 2 m^2^, 4 m^2^, 8 m^2^, 16 m^2^, 32 m^2^, 64 m^2^ and 128 m^2^ where each plot is nested within the next larger plot. We used the accumulative plots in order to construct the species accumulation curves (type I according to Scheiner^19^ typology) as well as the functional diversity accumulation curves.

### Trait selection and values

It is generally supported that correct estimates of functional diversity mainly depend on the choice of ecologically meaningful traits and that the selection is a difficult task and should depend on purposes of each study and the ecological processes to be studied^22^. The number of traits used is considered crucial for the calculation of the indices^42^. By including several traits one will enhance species functional uniqueness, whereas by using only a few traits one will enhance the probability of detecting redundancy of species^42^. Therefore, the number of traits included in the analysis must be adequate to capture the specific function of interest^42^. In our analysis the number of traits used seemed to be adequate to capture species functional uniqueness and avoid functional redundancy results due to a small traits number, since there was no significant variation resulted by using different number of traits each time. Moreover, the type of the traits, we believe that the wide range of functional characteristics used in our study contributed to the incorporate inter – among species but as well as among individuals –environment interactions.

For each recorded taxon we analyzed 26 functional traits. These traits represent vegetative characteristics, plant taxa ecological preferences (based on Ellenberg indices), and regenerative characteristics. They are related to the performance of species and represent different functional strategies for survival under particular environmental conditions. Traits representing vegetative characteristics include longevity, max plant height, mean leaf length, mean leaf width, leaf length/width ratio, life form, growth form, leaf surface texture and canopy structure. Plant taxa ecological preferences are based on Ellenberg’s indices and include indicators values for soil acidity, soil nutrient content, soil humidity, continentality, soil salt content, light and temperature. Regenerative characteristics include flowering period start, flowering period end, flowering period length, seed production, seed weight, flower size, flower sex, pollination type, fruit type and dispersal mode. Some of these traits are quantitative variables while others are categorical or ordinal. Trait values were collected from databases and bibliography, measured on plant specimens collected during field sampling or observed over several years in the field (the latter concerns flower phenology). For the seed weight of those species that no data could be found, we used genus values and in few cases the values were estimated from congeneric species with similar seed morphology. For detailed information about the type of the variables used, the classes of the categorical and ordinal variables and the sources of traits values see Karadimou *et al*.^6^ and Supplementary Table S1.

### Computing indices (FD, SD)

In our study we use trait-based functional diversity indices as functional diversity metrics. Although in some studies the term “functional diversity” is not regarded coherent to trait-based metrics^43^, in most of studies the term functional diversity is considered coherent to trait-based diversity (e.g. refs 4, 21 and 44). Therefore, although the term “traits diversity” is more accurate, in our study we use the term “functional diversity” to conform with the literature. We quantified the functional diversity of each community at each spatial scale using six multidimensional indices that explore different facets of functional diversity: functional richness (FRic), functional evenness (FEve), functional divergence (FDiv)^7^, functional dispersion (FDis)^9^, Rao’s quadratic entropy (RaoQ)^45^ and the dendrogram based FD index (FDen)^42^.

We calculated the five indices (FRic, FEve, FDiv, FDis and RaoQ) using the dbFD function of Lalibertè and Legendre^9^ FD package in R^46^. The function first uses Gower’s distance (to calculate multivariate distances between species based on the raw trait data). We chose the Gower’s distance because in our study we analyzed not only continuous variables (e.g. Ellenbergs indicators), but also qualitative functional traits (e.g. canopy structure) and circular traits (e.g. time of reproduction). To avoid negative eigenvalues, the Gower’s distance matrix is subject to square root transformation. These distance data are then subject to principal coordinates analysis (PCoA) and the resulting PCoA axes are used as new composite functional traits. As the indices require that there are more species than there are traits and because in certain scales and communities we have small numbers of species, we used only the first four PCoA axes. The use of the FD package allows us to have comparable results with previous studies exploring FDARs^16^. We calculated the FDen index with the ade4 package in R^46^.

Finally, we estimated Spearman’s rank correlation coefficients among the values of the indices to find out which of them are correlated.

Moreover, we calculated for each plot the values of three classical diversity indices: Simpson, Shannon and Pielou’s evenness with the ‘vegan’ package in R^46^.

One of the major questions on the estimation of FD is how many and which traits should be selected^20^. In order to investigate the effect of the functional traits group selected to compute functional diversity indices influences the indices values and moreover their relationship with area, we divided the initial traits data set into three categories according to three major groups referred above: vegetative characteristics group consisting of nine traits, plant taxa ecological preferences (based on Ellenberg indices) group consisting of seven traits, and regenerative characteristics group consisting of ten traits. Using these three data sets we repeated the above procedure to construct the functional diversity – area plots.

### Curve fitting & Evaluation of the goodness-of-fit

For the analysis we used the seven most commonly used models for species - area relationships: the power law, the logarithmic, the linear, the quadratic, the S-curve, the logistic and the Exponential models. We used nonlinear regression to fit the seven models to our data. To assess the goodness-of-fit of the seven models we used the adjusted coefficient of determinations 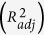 since we compare models with a different number of parameters^47^,^48^.

## Additional Information

**How to cite this article**: Karadimou, E. K. *et al*. Functional diversity exhibits a diverse relationship with area, even a decreasing one. *Sci. Rep.* **6**, 35420; doi: 10.1038/srep35420 (2016).

## Supplementary Material

Supplementary Information

## Figures and Tables

**Table 1:** Power model z values of the species accumulation curves (SAR), as well as the accumulation curves resulted from plotting the functional diversity’s indices FRic and FDen values vs a) area and b) species richness (SR) for the 16 plots used in this study.

z values - power model					
plots	SAR	FRic - area	FDen - area	FRic- SR	FDen-SR
1	0.124200	0.651600	0.10540	5.281000	0.84400
2	0.251500	0.199000	0.19120	0.827900	0.78830
3	0.243500	0.447200	0.19990	1.780900	0.78830
4	0.188300	0.322800	0.15270	1.794400	0.84470
5	0.143400	0.396200	0.13570	1.776400	0.61510
6	0.162900	0.540100	0.16870	2.944500	0.92880
7	0.220600	0.308100	0.17790	1.536300	0.87380
8	0.515000	0.539400	0.34730	1.224100	0.77640
9	0.515000	0.151500	0.12850	0.904900	0.80750
10	0.201400	0.367500	0.16470	1.976000	0.82290
11	0.114000	0.136800	0.08770	1.306000	0.79390
12	0.315400	0.535300	0.22920	1.635700	0.69940
13	0.123800	0.167800	0.08200	1.463500	0.76330
14	0.354300	1.634900	0.28610	4.516700	0.78210
15	0.132400	0.195300	0.13120	1.406100	0.94360
16	0.243200	0.277400	0.13780	1.724600	0.86150

**Table 2:** Power model statistics (adjusted R^2^ and *P* values) of the species accumulation curves (SAR) and the functional diversity accumulation curves (FDAR) for the 16 plots used in this study.

plots	Species richness - area		FRic - area		FDen - area		FDis - area		RaoQ - area		FEve - area		FDiv - area		Shannon - area		Simpson - area		Pielou’s evenness - area	
	R^2^	*P*	R^2^	*P*	R^2^	*P*	R^2^	*P*	R^2^	*P*	R^2^	*P*	R^2^	*P*	R^2^	*P*	R^2^	*P*	R^2^	*P*
1	0.954	0.000	0.854	0.001	0.907	0.000	0.424	0.080	0.668	0.069	0.873	0.001	0.572	0.030	0.529	0.41	0.340	0.129	0.332	0.135
2	0.992	0.000	0.981	0.000	0.092	0.000	0.584	0.027	0.618	0.021	0.872	0.001	0.617	0.021	0.814	0.002	0.358	0.117	0.977	0.000
3	0.977	0.000	0.738	0.006	0.926	0.000	0.743	0.006	0.708	0.009	0.975	0.000	0.610	0.022	0.094	0.461	0.714	0.008	0.934	0.000
4	0.970	0.000	0.771	0.004	0.928	0.000	0.922	0.000	0.932	0.000	0.857	0.001	0.001	0.937	0.425	0.080	0.009	0.818	0.720	0.008
5	0.986	0.000	0.945	0.000	0.950	0.000	0.829	0.002	0.813	0.002	0.746	0.006	0.659	0.014	0.739	0.006	0.148	0.346	0.103	0.439
6	0.992	0.000	0.640	0.017	0.717	0.008	0.614	0.021	0.359	0.116	0.721	0.008	0.395	0.095	0.742	0.006	0.601	0.024	0.242	0.216
7	0.992	0.000	0.886	0.000	0.970	0.000	0.000	0.970	0.007	0.841	0.580	0.028	0.934	0.000	0.608	0.022	0.000	0.960	0.943	0.000
8	0.977	0.000	0.909	0.000	0.944	0.000	0.133	0.375	0.120	0.401	0.517	0.045	0.208	0.255	0.957	0.000	0.883	0.001	0.050	0.594
9	0.954	0.000	0.944	0.000	0.895	0.000	0.864	0.001	0.851	0.001	0.796	0.003	0.757	0.005	0.322	0.142	0.556	0.034	0.367	0.111
10	0.964	0.000	0.740	0.006	0.962	0.000	0.005	0.862	0.006	0.861	0.852	0.001	0.003	0.900	0.438	0.074	0.024	0.712	0.781	0.004
11	0.927	0.000	0.705	0.009	0.855	0.001	0.008	0.828	0.005	0.871	0.853	0.001	0.178	0.297	0.862	0.001	0.800	0.003	0.533	0.040
12	0.800	0.003	0.950	0.000	0.962	0.000	0.730	0.007	0.388	0.099	0.607	0.023	0.151	0341	0456	0.066	0.413	0.86	0.962	0.000
13	0.959	0.000	0.848	0.001	0.974	0.000	0.213	0.250	0.079	0.501	0.682	0.012	0.491	0.053	0.797	0.003	0.612	0.022	0.101	0.443
14	0.985	0.000	0.834	0.002	0.982	0.000	0.794	0.003	0.811	0.002	0.037	0.684	0.564	0.032	0.812	0.078	0.401	0.092	0.003	0.895
15	0.977	0.000	0.920	0.000	0.942	0.000	0.219	0.243	0.384	0.101	0.848	0.076	0.725	0.040	0.918	0.000	0.892	0.000	0.470	0.060
16	0.944	0.000	0.970	0.000	0.979	0.000	0.888	0.000	0.850	0.001	0.605	0.023	0.010	0.815	0.102	0.442	0.003	0.892	0.941	0.000

We present only the results for the power law model for all diversity indices analyzed.
