# Supplementary material for: Functional diversity exhibits a diverse relationship with area, even a decreasing one
Source: Sci Rep. 2016 Oct 18;6:35420. doi: 10.1038/srep35420 (PMC5067660; doi:10.1038/srep35420)
Supplement: Supplementary Information [file srep35420-s1.pdf]

Functional diversity exhibits a diverse relationship with area, even a decreasing one.

Elpida K. Karadimou, Athanasios S. Kallimanis, Ioannis Tsiripidis and Panayotis Dimopoulos

Supplementary information

Supplementary Table S1. The 26 functional traits analyzed in this study. Sources for the information for each trait are identified as follows: (I) field observations, (II) plant specimens, (III) botanical descriptions, (IV) Royal Botanic Gardens Kew Seed Information Database (SID) (http://data.kew.org/sid/, assessed September 1st, 2013), (V) Baseflore (http://perso.wanadoo.fr/philippe.julve/catminat.htm, accessed September 1st, 2013), (VI) Pignatti*et al*. (2005) and (VII) Böhling*et al*. (2002). Cat: 1: vegetative characteristics, 2: plant taxa ecological preferences (based on Ellenberg indices), and 3: regenerative characteristics.

Cat.	Functional trait	Type of variable	Classes	Sources
1	Longevity	Categorical	Annual; Perennial	III, VI
1	Max plant height	Quantitative (mm)	-	II, III
1	Mean leaf length	Quantitative (mm)	-	II, III
1	Mean leaf width	Quantitative (mm)	-	II, III
1	Leaf length/ width ratio	Quantitative (mm)	-	calculated
1	Life form	Categorical	Chamaephyte; Geophyte; Phanerophyte; Therophyte; Hemicryptophyte	III, VI
1	Growth form	Categorical	Bulbose; Caespitose; Fruticose; Parasite; Reptant	III, VI

			Rosulate; Scapose	
1	Leaf surface texture	Categorical	Glabrous; Pubescent	II, III
1	Canopy structure	Categorical	No leaves; basal leaves; leafy stem; parasite; semibasal (according to Hödgson et al., 1995)	II, III
2	Ellenberg's indicators values for soil acidity, soil nutrient content, soil humidity, continentality, soil salt content, light, temperature	Ordinal	-	VII
3	Flowering period start	treated as quantitative	-	I, III
3	Flowering period end	treated as quantitative	-	I, III
3	Flowering period length	treated as quantitative	-	I, III
3	Seed production	Ordinal	Low (< 25 seeds per plant); Medium (25 – 100 seeds per plant); High (> 100 seeds per plant)	I, III
3	Seed weight	Quantitative (g)	-	IV
3	Flower size	Categorical	Big (>10 mm); Medium (5-10 mm); Small (<5 mm); No flower (Graminae)	III
3	Flower sex	Categorical	Monosexual; Hermaphrodite	V
3	Pollination type	Categorical	Anemogamy; Autogamy; Entomogamy; Hydrogamy	V
3	Fruit type	Categorical	Achene; Berry; Capsule; Caryopsis; Follicle; Legume; Siliqula; Spores	III, V
3	Dispersal mode	Categorical	Anemochory; Zoochory; Hydrochory; Barochory	III, V

Supplementary Table S2. Adjusted R square and *P* values of the seven models examined: Linear, Logarithmic, Quadratic, Power, Sigmoid (S) and Logistic, for the functional diversity accumulation curve (FDAC) of each of the six functional diversity indices. Plots 1, 6, 7, 16 belong to *Lupinus angustifolius* - *Hyparrhenia hirta* community, plots 2, 3, 4, 5 belong to *Lupinus angustifolius* - *Helichrysum italicum* community, plots 8, 10, 12, 14 to *Pistacia lentiscus* community and plots 9, 11, 13, 15 to *Lupinus angustifolius* - *Tolpis barbata* community.

	R^2_{adj}										*P* values									
FD Indices	FRic	FDen	SR	FDis	RaoQ	FEve	FDiv	Shannon	Simpson	Pielou's evenness	FRic	FDen	SR	FDis	RaoQ	FEve	FDiv	Shannon	Simpson	Pielou's evenness
plot 1																				
Linear	0.522	0.550	0.500	0.700	0.817	0.752	0.081	0.776	0.637	-0.116	0.028	0.021	0.030	0.006	0.001	0.003	0.253	0.002	0.011	0.612
Logarithmic	0.850	0.895	0.965	0.349	0.612	0.838	0.526	0.462	0.258	0.250	0.001	0.000	0.000	0.072	0.013	0.001	0.026	0.038	0.113	0.118
Quadratic	0.810	0.826	0.811	0.655	0.862	0.928	0.354	0.765	0.568	0.027	0.007	0.005	0.007	0.030	0.003	0.001	0.147	0.012	0.053	0.402
Power	0.830	0.891	0.947	0.328	0.613	0.852	0.500	0.451	0.230	0.220	0.001	0.000	0.000	0.080	0.013	0.001	0.031	0.041	0.129	0.135
S	0.761	0.618	0.800	-0.088	0.075	0.369	0.473	-0.016	-0.115	0.495	0.003	0.013	0.002	0.535	0.257	0.065	0.036	0.381	0.618	0.031
Exponential	0.357	0.511	0.444	0.647	0.804	0.733	0.063	0.764	0.605	-0.126	0.069	0.028	0.043	0.008	0.002	0.004	0.271	0.003	0.014	0.658
Logistic	0.357	0.511	0.444	0.647	0.804	0.733	0.063	0.764	0.605	-0.126	0.069	0.028	0.043	0.008	0.002	0.004	0.271	0.003	0.014	0.658
plot 2																				
Linear	0.715	0.743	0.778	0.118	0.145	0.663	0.100	0.218	-0.140	0.607	0.005	0.004	0.002	0.215	0.190	0.008	0.231	0.137	0.721	0.014
Logarithmic	0.980	0.987	0.987	0.559	0.586	0.863	0.541	0.787	0.250	0.972	0.000	0.000	0.000	0.020	0.016	0.001	0.023	0.002	0.118	0.000
Quadratic	0.828	0.890	0.905	0.225	0.234	0.969	0.170	0.610	0.340	0.829	0.005	0.002	0.001	0.228	0.221	0.000	0.271	0.041	0.153	0.005
Power	0.957	0.990	0.991	0.553	0.554	0.851	0.515	0.783	0.251	0.973	0.000	0.000	0.000	0.021	0.021	0.001	0.027	0.002	0.117	0.000
S	0.819	0.726	0.761	0.709	0.883	0.312	0.892	0.725	0.490	0.601	0.001	0.004	0.003	0.005	0.000	0.087	0.000	0.005	0.032	0.015
Exponential	0.555	0.619	0.607	0.117	0.118	0.695	0.081	0.215	-0.139	0.633	0.021	0.013	0.014	0.215	0.213	0.006	0.251	0.139	0.718	0.011
Logistic	0.555	0.619	0.607	0.117	0.118	0.695	0.081	0.215	-0.139	0.633	0.021	0.013	0.014	0.215	0.213	0.006	0.251	0.139	0.718	0.011
plot 3																				
Linear	0.563	0.556	0.642	0.474	0.399	0.530	0.725	0.085	0.633	0.667	0.019	0.020	0.010	0.035	0.055	0.024	0.005	0.246	0.011	0.008
Logarithmic	0.957	0.965	0.982	0.700	0.659	0.966	0.559	-0.061	0.670	0.929	0.000	0.000	0.000	0.006	0.009	0.000	0.020	0.469	0.008	0.000
Quadratic	0.792	0.817	0.920	0.790	0.694	0.833	0.817	-0.097	0.683	0.901	0.008	0.006	0.001	0.009	0.022	0.005	0.006	0.544	0.024	0.001
Power	0.694	0.913	0.973	0.700	0.659	0.970	0.545	-0.058	0.667	0.923	0.006	0.000	0.000	0.006	0.009	0.000	0.023	0.461	0.008	0.000
S	0.958	0.878	0.782	0.354	0.421	0.686	-0.026	-0.160	0.279	0.501	0.000	0.000	0.002	0.070	0.048	0.007	0.399	0.858	0.103	0.030
Exponential	0.217	0.428	0.518	0.473	0.395	0.588	0.700	0.086	0.642	0.701	0.137	0.047	0.027	0.036	0.056	0.016	0.006	0.245	0.010	0.006
Logistic	0.217	0.428	0.518	0.473	0.395	0.588	0.700	0.086	0.642	0.701	0.137	0.047	0.027	0.036	0.056	0.016	0.006	0.245	0.010	0.006
plot 4																				

Linear	0.733	0.545	0.663	0.549	0.481	0.344	-0.109	0.588	-0.119	0.198	0.004	0.022	0.009	0.022	0.034	0.075	0.619	0.016	0.630	0.149
Logarithmic	0.921	0.953	0.984	0.908	0.923	0.796	-0.165	0.332	-0.154	0.652	0.000	0.000	0.000	0.000	0.000	0.002	0.913	0.079	0.805	0.009
Quadratic	0.739	0.661	0.786	0.897	0.813	0.441	-0.323	0.508	-0.173	0.436	0.015	0.029	0.009	0.001	0.007	0.102	0.882	0.073	0.643	0.103
Power	0.892	0.916	0.965	0.909	0.920	0.833	-0.165	0.330	-0.156	0.673	0.000	0.000	0.000	0.000	0.000	0.001	0.917	0.080	0.818	0.008
S	0.860	0.862	0.796	0.563	0.674	0.884	-0.032	0.026	-0.110	0.510	0.001	0.001	0.002	0.019	0.008	0.000	0.411	0.318	0.601	0.028
Exponential	0.468	0.440	0.533	0.546	0.474	0.398	-0.108	0.572	-0.113	0.210	0.037	0.043	0.024	0.022	0.035	0.055	0.954	0.018	0.612	0.142
Logistic	0.468	0.440	0.533	0.546	0.474	0.398	-0.108	0.572	-0.113	0.210	0.037	0.043	0.024	0.022	0.035	0.055	0.954	0.018	0.612	0.142
plot 5																				
Linear	0.663	0.548	0.657	0.385	0.372	0.704	0.259	0.696	0.358	-0.166	0.009	0.022	0.009	0.064	0.064	0.006	0.113	0.006	0.069	0.972
Logarithmic	0.948	0.962	0.992	0.803	0.789	0.896	0.598	0.498	0.040	0.016	0.000	0.000	0.000	0.002	0.002	0.000	0.015	0.030	0.300	0.332
Quadratic	0.911	0.787	0.879	0.625	0.620	0.943	0.425	0.714	0.232	-0.233	0.001	0.009	0.002	0.038	0.038	0.000	0.108	0.019	0.223	0.728
Power	0.936	0.941	0.983	0.800	0.782	0.878	0.602	0.377	0.006	-0.047	0.000	0.000	0.000	0.002	0.002	0.000	0.014	0.062	0.346	0.439
S	0.709	0.709	0.755	0.506	0.486	0.383	0.307	-0.076	-0.161	0.423	0.005	0.005	0.003	0.029	0.033	0.060	0.089	0.504	0.872	0.048
Exponential	0.487	0.480	0.571	0.379	0.361	0.755	0.262	0.511	0.247	-0.159	0.033	0.034	0.018	0.061	0.067	0.003	0.111	0.028	0.119	0.848
Logistic	0.487	0.480	0.571	0.379	0.361	0.755	0.262	0.511	0.247	-0.159	0.033	0.034	0.018	0.061	0.067	0.003	0.111	0.028	0.119	0.848
plot 6																				
Linear	0.580	0.670	0.742	0.592	0.429	0.495	0.071	0.285	0.108	-0.133	0.017	0.008	0.004	0.016	0.046	0.031	0.261	0.099	0.223	0.688
Logarithmic	0.906	0.933	0.958	0.548	0.242	0.712	0.310	0.752	0.559	0.085	0.000	0.000	0.000	0.022	0.122	0.005	0.088	0.003	0.020	0.246
Quadratic	0.885	0.940	0.967	0.591	0.333	0.827	-0.092	0.428	0.200	-0.215	0.002	0.000	0.000	0.046	0.157	0.005	0.537	0.090	0.247	0.701
Power	0.852	0.946	0.981	0.550	0.253	0.675	0.294	0.699	0.535	0.115	0.001	0.000	0.000	0.021	0.116	0.008	0.095	0.006	0.024	0.216
S	0.813	0.584	0.607	0.181	-0.046	0.243	0.527	0.914	0.881	0.550	0.001	0.017	0.014	0.161	0.438	0.122	0.025	0.000	0.000	0.021
Exponential	0.373	0.608	0.670	0.600	0.442	0.460	0.067	0.223	0.087	-0.124	0.063	0.014	0.008	0.015	0.043	0.039	0.266	0.133	0.244	0.649
Logistic	0.373	0.608	0.670	0.600	0.442	0.460	0.067	0.223	0.087	-0.124	0.063	0.014	0.008	0.015	0.043	0.039	0.266	0.133	0.244	0.649
plot 7																				
Linear	0.567	0.724	0.765	-0.018	0.058	0.068	0.598	-0.015	0.077	0.710	0.019	0.005	0.003	0.386	0.277	0.266	0.015	0.380	0.255	0.005
Logarithmic	0.869	0.959	0.977	-0.167	-0.159	0.526	0.925	0.534	-0.166	0.640	0.000	0.000	0.000	0.978	0.844	0.025	0.000	0.024	0.955	0.000
Quadratic	0.872	0.933	0.917	-0.084	-0.005	0.283	0.849	0.227	0.106	0.881	0.003	0.000	0.001	0.527	0.437	0.188	0.004	0.113	0.326	0.002
Power	0.868	0.965	0.990	-0.166	-0.158	0.510	0.922	0.543	-0.166	0.933	0.000	0.000	0.000	0.970	0.841	0.028	0.000	0.022	0.960	0.000
S	0.467	0.554	0.698	-0.118	-0.131	0.482	0.640	0.883	0.065	0.582	0.037	0.021	0.006	0.629	0.678	0.034	0.010	0.000	0.269	0.017
Exponential	0.501	0.638	0.632	-0.013	0.060	0.049	0.556	-0.007	0.080	0.744	0.030	0.011	0.011	0.378	0.274	0.288	0.020	0.368	0.251	0.004
Logistic	0.501	0.638	0.632	-0.013	0.060	0.049	0.556	-0.007	0.080	0.744	0.030	0.011	0.011	0.378	0.274	0.288	0.020	0.368	0.251	0.004
plot 8																				
Linear	0.735	0.699	0.716	0.315	0.292	0.205	0.472	0.421	0.399	-0.045	0.004	0.006	0.005	0.086	0.096	0.145	0.036	0.049	0.055	0.435
Logarithmic	0.935	0.939	0.962	0.013	-0.008	0.443	0.075	0.908	0.868	-0.110	0.000	0.000	0.000	0.336	0.369	0.043	0.257	0.000	0.000	0.600
Quadratic	0.893	0.925	0.918	0.180	0.151	0.328	0.372	0.696	0.662	-0.250	0.002	0.001	0.002	0.263	0.286	0.159	0.135	0.022	0.029	0.754
Power	0.894	0.935	0.947	-0.012	-0.027	0.436	0.077	0.901	0.864	-0.108	0.000	0.000	0.000	0.375	0.401	0.045	0.255	0.000	0.001	0.594
S	0.591	0.588	0.780	-0.158	-0.153	0.154	-0.166	0.727	0.642	-0.143	0.016	0.016	0.002	0.839	0.801	0.183	0.966	0.004	0.010	0.739
Exponential	0.484	0.550	0.469	0.217	0.202	0.200	0.475	0.403	0.390	-0.044	0.033	0.021	0.037	0.137	0.147	0.148	0.035	0.054	0.058	0.434
Logistic	0.484	0.550	0.469	0.217	0.202	0.200	0.475	0.403	0.390	-0.044	0.033	0.021	0.037	0.137	0.147	0.148	0.035	0.054	0.058	0.434

plot 9																				
Linear	0.521	0.367	0.716	0.358	0.383	0.257	0.434	0.209	0.030	-0.096	0.026	0.065	0.005	0.069	0.060	0.114	0.045	0.142	0.312	0.557
Logarithmic	0.945	0.911	0.962	0.844	0.829	0.781	0.711	0.730	0.496	0.260	0.000	0.000	0.000	0.001	0.001	0.002	0.005	0.004	0.031	0.112
Quadratic	0.817	0.667	0.918	0.646	0.675	0.599	0.660	0.347	0.077	-0.145	0.006	0.028	0.001	0.032	0.026	0.044	0.029	0.149	0.353	0.605
Power	0.934	0.877	0.947	0.842	0.826	0.762	0.716	0.695	0.482	0.261	0.000	0.000	0.000	0.001	0.001	0.003	0.005	0.006	0.034	0.111
S	0.696	0.896	0.780	0.647	0.560	0.599	0.264	0.994	0.956	0.838	0.006	0.000	0.002	0.010	0.020	0.015	0.110	0.000	0.000	0.001
Exponential	0.464	0.316	0.469	0.355	0.378	0.249	0.440	0.182	0.024	-0.094	0.038	0.085	0.037	0.070	0.062	0.188	0.044	0.161	0.320	0.550
Logistic	0.464	0.316	0.469	0.355	0.378	0.249	0.440	0.182	0.024	-0.094	0.038	0.085	0.037	0.070	0.062	0.188	0.044	0.161	0.320	0.550
plot 10																				
Linear	0.615	0.672	0.608	-0.165	-0.165	0.517	-0.166	0.120	-0.065	0.370	0.013	0.008	0.014	0.925	0.934	0.027	0.957	0.212	0.921	0.065
Logarithmic	0.935	0.978	0.992	-0.163	-0.163	0.843	-0.163	0.351	-0.146	0.738	0.000	0.000	0.000	0.901	0.900	0.001	0.895	0.071	0.751	0.004
Quadratic	0.773	0.843	0.849	-0.287	-0.285	0.931	-0.306	-0.048	-0.307	0.812	0.019	0.004	0.006	0.811	0.806	0.001	0.580	0.615	0.612	0.005
Power	0.697	0.955	0.958	-0.160	-0.160	0.828	-0.163	0.344	-0.138	0.744	0.006	0.000	0.000	0.862	0.861	0.001	0.900	0.074	0.712	0.004
S	0.920	0.824	0.838	0.063	0.060	0.393	0.029	0.688	0.125	0.370	0.000	0.001	0.001	0.271	0.274	0.057	0.314	0.007	0.207	0.064
Exponential	0.260	0.547	0.483	-0.166	-0.166	0.516	-0.166	0.099	-0.163	0.379	0.112	0.022	0.033	0.949	0.658	0.027	0.954	0.232	0.898	0.062
Logistic	0.260	0.547	0.483	-0.166	-0.166	0.516	-0.166	0.099	-0.163	0.379	0.112	0.022	0.033	0.949	0.658	0.027	0.954	0.232	0.898	0.062
plot 11																				
Linear	0.142	0.313	0.461	0.065	-0.051	0.258	-0.167	0.825	0.822	0.715	0.192	0.087	0.038	0.268	0.447	0.113	0.995	0.001	0.001	0.005
Logarithmic	0.686	0.860	0.950	-0.157	-0.161	0.824	0.049	0.850	0.807	0.531	0.007	0.001	0.000	0.835	0.866	0.001	0.288	0.001	0.002	0.024
Quadratic	0.314	0.541	0.700	-0.061	-0.150	0.644	-0.223	0.971	0.955	0.757	0.168	0.062	0.021	0.500	0.611	0.033	0.713	0.000	0.001	0.048
Power	0.656	0.831	0.914	-0.157	-0.161	0.829	0.042	0.839	0.766	0.455	0.009	0.001	0.000	0.828	0.871	0.001	0.297	0.001	0.003	0.040
S	0.873	0.897	0.892	-0.131	-0.046	0.769	0.513	0.291	0.199	-0.066	0.000	0.000	0.000	0.677	0.438	0.438	0.003	0.097	0.149	0.481
Exponential	0.121	0.278	0.394	0.068	-0.050	0.270	-0.167	0.684	0.670	0.580	0.210	0.103	0.056	0.265	0.445	0.445	0.107	0.007	0.008	0.017
Logistic	0.121	0.278	0.394	0.068	-0.050	0.270	-0.167	0.684	0.670	0.580	0.210	0.103	0.056	0.265	0.445	0.445	0.107	0.007	0.008	0.017
plot 12																				
Linear	0.697	0.702	0.767	0.685	0.685	0.280	0.212	-0.090	0.315	0.608	0.006	0.006	0.003	0.007	0.007	0.102	0.141	0.539	0.086	0.014
Logarithmic	0.890	0.944	0.939	0.350	0.348	0.536	0.009	0.359	-0.056	0.955	0.000	0.000	0.000	0.072	0.072	0.024	0.342	0.068	0.457	0.000
Quadratic	0.979	0.955	0.978	0.683	0.982	0.392	0.091	0.089	0.203	0.789	0.000	0.000	0.000	0.024	0.025	0.124	0.339	0.168	0.903	0.015
Power	0.942	0.956	0.979	0.288	0.285	0.541	0.009	0.365	-0.054	0.956	0.000	0.000	0.000	0.098	0.099	0.023	0.341	0.066	0.454	0.000
S	0.621	0.678	0.711	-0.135	-0.136	0.443	-0.163	0.577	-0.166	0.733	0.012	0.007	0.005	0.697	0.703	0.043	0.901	0.018	0.958	0.004
Exponential	0.549	0.593	0.616	0.616	0.615	0.288	0.212	-0.087	0.318	0.624	0.022	0.015	0.013	0.013	0.013	0.098	0.140	0.532	0.085	0.012
Logistic	0.549	0.593	0.616	0.616	0.615	0.288	0.212	-0.087	0.318	0.624	0.022	0.015	0.013	0.013	0.013	0.098	0.140	0.532	0.085	0.012
plot 13																				
Linear	0.926	0.607	0.548	-0.150	-0.165	0.129	-0.061	0.660	0.377	-0.166	0.000	0.014	0.022	0.780	0.928	0.203	0.469	0.009	0.062	0.945
Logarithmic	0.695	0.985	0.979	0.084	-0.073	0.617	0.408	0.763	0.552	-0.046	0.006	0.000	0.000	0.248	0.496	0.013	0.052	0.003	0.021	0.438
Quadratic	0.916	0.792	0.789	-0.082	-0.177	0.221	0.024	0.722	0.350	-0.098	0.001	0.008	0.011	0.525	0.648	0.231	0.405	0.049	0.213	0.544
Power	0.823	0.970	0.952	0.082	-0.075	0.629	0.406	0.763	0.547	-0.049	0.001	0.000	0.000	0.250	0.501	0.012	0.053	0.003	0.022	0.443
S	0.362	0.805	0.842	0.140	-0.030	0.923	0.908	0.503	0.577	-0.038	0.067	0.002	0.001	0.194	0.407	0.000	0.000	0.029	0.018	0.422
Exponential	0.890	0.548	0.471	-0.151	-0.164	0.141	-0.063	0.639	0.363	-0.166	0.000	0.022	0.036	0.786	0.918	0.193	0.474	0.011	0.067	0.957

Logistic	0.890	0.548	0.471	-0.151	-0.164	0.141	-0.063	0.639	0.363	-0.166	0.000	0.022	0.036	0.786	0.918	0.193	0.474	0.011	0.067	0.957
plot 14																				
Linear	0.785	0.693	0.741	0.813	0.815	0.079	0.628	0.780	0.696	0.059	0.002	0.006	0.004	0.001	0.001	0.252	0.012	0.002	0.006	0.275
Logarithmic	0.937	0.988	0.972	0.806	0.819	-0.124	0.491	0.557	0.363	-0.164	0.000	0.000	0.000	0.002	0.001	0.651	0.032	0.020	0.067	0.918
Quadratic	0.951	0.923	0.954	0.951	0.955	-0.083	0.633	0.852	0.696	-0.106	0.000	0.001	0.000	0.000	0.000	0.526	0.035	0.016	0.094	0.989
Power	0.806	0.979	0.982	0.760	0.779	-0.123	0.492	0.522	0.301	-0.163	0.002	0.000	0.000	0.003	0.002	0.648	0.032	0.026	0.092	0.895
S	0.932	0.763	0.761	0.194	0.219	-0.097	-0.037	-0.010	-0.130	0.055	0.000	0.003	0.003	0.152	0.136	0.560	0.419	0.372	0.674	0.280
Exponential	0.253	0.537	0.556	0.662	0.664	0.081	0.630	0.733	0.628	0.069	0.116	0.023	0.021	0.009	0.008	0.251	0.011	0.004	0.012	0.264
Logistic	0.253	0.537	0.556	0.662	0.664	0.081	0.630	0.733	0.628	0.069	0.116	0.023	0.021	0.009	0.008	0.251	0.011	0.004	0.012	0.264
plot 15																				
Linear	0.426	0.550	0.601	-0.008	0.194	0.387	0.259	0.852	0.845	0.738	0.047	0.021	0.015	0.369	0.152	0.059	0.113	0.001	0.001	0.004
Logarithmic	0.917	0.931	0.990	0.089	0.289	0.857	0.804	0.881	0.857	0.396	0.000	0.000	0.000	0.242	0.098	0.001	0.002	0.000	0.001	0.056
Quadratic	0.770	0.875	0.852	-0.152	0.122	0.788	0.614	0.972	0.948	0.699	0.011	0.002	0.005	0.614	0.312	0.009	0.040	0.000	0.000	0.021
Power	0.907	0.932	0.974	0.088	0.282	0.845	0.808	0.904	0.874	0.382	0.000	0.000	0.000	0.243	0.101	0.001	0.001	0.000	0.000	0.060
S	0.795	0.622	0.796	-0.027	0.038	0.539	0.824	0.440	0.431	-0.036	0.002	0.012	0.002	0.402	0.301	0.023	0.001	0.043	0.046	0.418
Exponential	0.373	0.511	0.522	-0.009	0.185	0.399	0.264	0.819	0.816	0.716	0.063	0.028	0.026	0.371	0.159	0.055	0.110	0.001	0.001	0.005
Logistic	0.373	0.511	0.522	-0.009	0.185	0.399	0.264	0.819	0.816	0.716	0.063	0.028	0.026	0.371	0.159	0.055	0.110	0.001	0.001	0.005
plot 16																				
Linear	0.705	0.623	0.604	0.815	0.829	0.869	-0.093	-0.048	0.015	0.830	0.006	0.012	0.014	0.001	0.001	0.000	0.550	0.442	0.333	0.001
Logarithmic	0.941	0.982	0.988	0.859	0.816	0.538	-0.158	0.449	-0.163	0.941	0.000	0.000	0.000	0.001	0.001	0.023	0.840	0.041	0.892	0.000
Quadratic	0.951	0.870	0.813	0.903	0.880	0.847	-0.307	0.044	-0.060	0.937	0.000	0.003	0.007	0.001	0.002	0.004	0.843	0.209	0.663	0.000
Power	0.965	0.976	0.935	0.869	0.825	0.539	-0.155	0.458	-0.163	0.931	0.000	0.000	0.000	0.000	0.001	0.023	0.815	0.039	0.892	0.000
S	0.631	0.722	0.873	0.483	0.444	0.040	0.170	0.859	0.102	0.496	0.011	0.005	0.000	0.033	0.042	0.299	0.170	0.001	0.229	0.031
Exponential	0.593	0.552	0.447	0.784	0.795	0.899	-0.099	-0.043	0.016	0.849	0.015	0.021	0.042	0.002	0.002	0.000	0.567	0. 431	0.332	0.001
Logistic	0.593	0.552	0.447	0.784	0.795	0.899	-0.099	-0.043	0.016	0.849	0.015	0.021	0.042	0.002	0.002	0.000	0.567	0. 431	0.332	0.001

Supplementary Table S3. R square and *P* values of the six models examined: Linear, Logarithmic, Quadratic, Power, Sigmoid (S), Exponential and Logistic, for the functional diversity area relationship (FDAR) of each of the six functional diversity indices. Plots 1, 6, 7, 16 belong to *Lupinus angustifolius* - *Hyparrhenia hirta* community, plots 2, 3, 4, 5 belong to *Lupinus angustifolius* - *Helichrysum italicum* community, plots 8, 10, 12, 14 to *Pistacia lentiscus* community and plots 9, 11, 13, 15 to *Lupinus angustifolius* - *Tolpis barbata* community.

	**R^2_{adj}**										***P* values**									
FD Indices	**FRic**	**FDen**	**SR**	**FDis**	**RaoQ**	**FEve**	**FDiv**	**Shannon**	**Simpson**	**Pielou's evenness**	**FRic**	**FDen**	**SR**	**FDis**	**RaoQ**	**FEve**	**FDiv**	**Shannon**	**Simpson**	**Pielou's evenness**
**plot 1**																				
Linear	-0,059	0.077	0.105	0.152	0.122	-0.008	-0.058	0.172	0.167	-0.022	0,739	0.147	0.111	0.068	0.093	0.365	0.728	0.055	0.053	0.430
Logarithmic	0.032	0.245	0.285	0.453	0.475	-0.023	-0.043	0.503	0.467	0.068	0.236	0.025	0.016	0.002	0.001	0.436	0.571	0.001	0.001	0.162
Quadratic	-0.085	0.114	0.153	0.305	0.267	-0.077	-0.132	0.346	0.328	-0.041	0.964	0.168	0.123	0.031	0.045	0.660	0.936	0.027	0.024	0.520
Power	0.083	0.247	0.281	0.453	0.498	-0.016	-0.043	0.477	0.433	0.	0.138	0.025	0.017	0.002	0.001	0.402	0.565	0.001	0.002	0.151
S	0.197	0.281	0.309	0.515	0.660	-0.049	-0.037	0.568	0.469	0.112	0.042	0.017	0.012	0.001	0.000	0.612	0.522	0.000	0.001	0.103
Exponential	-0.43	0.073	0.096	0.148	0.127	-0.003	-0.054	0.154	0.155	-0.017	0.570	0.153	0.112	0.071	0.088	0.346	0.674	0.067	0.066	0.406.
Logistic	-0.43	0.073	0.096	0.148	0.127	-0.003	-0.054	0.154	0.155	-0.017	0.570	0.153	0.112	0.071	0.088	0.346	0.674	0.067	0.066	0.406.
**plot 2**																				
Linear	0.064	0.518	0.563	-0.013	0.003	-0.001	-0.016	0.014	-0.066	-0.041	0.168	0.001	0.000	0.385	0.323	0.337	0.398	0.286	0.942	0.553
Logarithmic	0.318	0.808	0.824	0.008	0.051	-0.067	0.080	0.059	-0.140	0.003	0.011	0.000	0.000	0.304	0.193	0.969	0.143	0.177	0.983	0.324
Quadratic	0.144	0.681	0.708	-0.047	-0.011	-0.015	-0.049	0.015	-0.067	-0.109	0.132	0.000	0.000	0.541	0.423	0.437	0.549	0.354	0.978	0.811
Power	0.267	0.714	0.712	-0.001	0.041	-0.067	0.092	0.035	-0.059	-0.020	0.020	0.000	0.000	0.338	0.214	0.984	0.126	0.227	0.752	0.420
S	0.382	0.689	0.678	-0.029	0.014	-0.031	0.150	-0.002	-0.065	0.016	0.005	0.000	0.000	0.470	0.284	0.482	0.069	0.342	0.876	0.280
Exponential	0.037	0.364	0.373	-0.017	0.000	-0.003	-0.011	0.005	-0.065	-0.053	0.222	0.006	0.005	0.404	0.334	0.343	0.376	0.317	0.876	0.664
Logistic	0.037.	0.364	0.373	-0.017	0.000	-0.003	-0.011	0.005	-0.065	-0.053	0.222	0.006	0.005	0.404	0.334	0.343	0.376	0.317	0.876	0.664
**plot 3**																				
Linear	0.015	0.192	0.238	0.072	0.051	-0.061	0.267	0.015	0.205	0.288	0.283	0.045	0.027	0.156	0.193	0.784	0.020	0.282	0.039	0.015
Logarithmic	0.303	0.597	0.709	0.148	0.107	-0.066	0.657	-0.032	0.315	0.526	0.013	0.000	0.000	0.071	0.108	0.903	0.000	0.493	0.011	0.001
Quadratic	0.151	0.414	0.542	0.154	0.115	-0.082	0.603	-0.018	0.387	0.580	0.125	0.009	0.002	0.122	0.167	0.681	0.001	0.446	0.013	0.001

Power	0.235	0.571	0.679	0.147	0.113	-0.066	0.641	-0.020	0.307	0.497	0.028	0.000	0.000	0.072	0.101	0.901	0.000	0.421	0.012	0.001
S	0.376	0.693	0.814	0.075	0.041	-0.061	0.639	-0.066	0.169	0.356	0.005	0.000	0.000	0.150	0.214	0.780	0.000	0.919	0.057	0.007
Exponential	0.005	0.172	0.208	0.065	0.050	-0.061	0.248	0.012	0.182	0.263	0.317	0.055	0.038	0.167	0.195	0.776	0.024	0.291	0.050	0.021
Logistic	0.005	0.172	0.208	0.065	0.050	-0.061	0.248.	0.012	0.182	0.263	0.317	0.055	0.038	0.167	0.195	0.776	0.024	0.291	0.050	0.021
**plot 4**																				
Linear	0.635	0.619	0.651	-0.028	-0.050	0.016	-0.027	-0.049	0.151	0.260	0.000	0.000	0.000	0.462	0.632	0.280	0.460	0.619	0.069	0.021
Logarithmic	0.535	0.778	0.800	-0.051	-0.065	0.173	0.131	-0.016	0.333	0.478	0.001	0.000	0.000	0.641	0.882	0.054	0.084	0.400	0.009	0.001
Quadratic	0.609	0.668	0.760	-0.055	-0.123	0.026	0.232	0.088	0.451	0.556	0.001	0.000	0.000	0.572	0.885	0.327	0.062	0.206	0.006	0.001
Power	0.523	0.757	0.763	-0.050	-0.065	0.169	0.131	-0.009	0.328	0.470	0.001	0.000	0.000	0.637	0.871	0.057	0.085	0.370	0.010	0.001
S	0.356	0.647	0.583	-0.064	-0.002	0.250	0.143	-0.044	0.209	0.318	0.007	0.000	0.000	0.845	0.342	0.024	0.075	0.576	0.037	0.011
Exponential	0.552	0.524	0.539	-0.029	-0.052	0.014	-0.026	-0.050	0.140	0.250	0.000	0.001	0.000	0.469	0.651	0.286	0.453	0.631	0.077	0.024
Logistic	0.552	0.524	0.539	-0.029	-0.052	0.014	-0.026	-0.050	0.140	0.250	0.000	0.001	0.000	0.469	0.651	0.286	0.453	0.631	0.077	0.024
**plot 5**																				
Linear	-0.023	0.141	0.114	-0.067	-0.047	0.082	0.227	0.082	0.225	0.294	0.437	0.076	0.101	0.977	0.602	0.141	0.031	0.140	0.031	0.014
Logarithmic	0.049	0.199	0.139	0.098	0.193	0.108	0.006	-0.066	-0.017	0.061	0.196	0.041	0.078	0.119	0.044	0.107	0.311	0.959	0.407	0.173
Quadratic	-0.037	0.167	0.129	0.437	0.469	0.165	0.194	0.278	0.295	0.274	0.506	0.101	0.149	0.007	0.005	0.112	0.087	0.040	0.034	0.042
Power	0.020	0.199	0.157	0.091	0.181	0.113	0.008	-0.066	-0.020	0.042	0.267	0.042	0.064	0.128	0.050	0.102	0.306	0.918	0.422	0.213
S	0.41	0.120	0.068	0.204	0.264	0.003	-0.066	-0.022	-0.065	-0.060	0.215	0.094	0.162	0.039	0.020	0.322	0.948	0.433	0.896	0.765
Exponential	-0.038	0.124	0.110	-0.067	-0.047	0.083	0.226	0.094	0.213	0.268	0.529	0.091	0.105	0.981	0.604	0.138	0.031	0.124	0.036	0.019
Logistic	-0.038	0.124	0.110	-0.067	-0.047	0.083	0.226	0.094	0.213	0.268	0.529	0.091	0.105	0.981	0.604	0.138	0.031	0.124	0.036	0.019
**plot 6**																				
Linear	0.218	0.304	0.326	-0.038	-0.040	-0.016	-0.045	-0.066	-0.036	0.005	0.034	0.013	0.010	0.532	0.545	0.401	0.584	0.936	0.514	0.316
Logarithmic	0.399	0.719	0.749	0.185	0.155	0.286	0.115	0.047	0.226	0.356	0.004	0.000	0.000	0.048	0.066	0.016	0.099	0.200	0.031	0.007
Quadratic	0.345	0.736	0.755	0.021	0.010	0.308	-0.054	-0.070	0.051	0.181	0.020	0.000	0.000	0.338	0.365	0.030	0.569	0.629	0.272	0.097
Power	0.303	0.699	0.726	0.166	0.141	0.282	0.083	0.038	0.198	0.325	0.013	0.000	0.000	0.059	0.077	0.016	0.138	0.221	0.042	0.010
S	0.272	0.662	0.689	0.414	0.355	0.443	0.275	0.256	0.483	0.613	0.018	0.000	0.000	0.003	0.007	0.002	0.018	0.022	0.001	0.000
Exponential	0.129	0.279	0.297	-0.046	-0.048	-0.018	-0.055	-0.067	-0.045	-0.009	0.087	0.017	0.014	0.595	0.615	0.410	0.686	0.665	0.583	0.369
Logistic	0.129	0.279	0.297	-0.046	-0.048	-0.018	-0.055	-0.067	-0.045	-0.009	0.087	0.017	0.014	0.595	0.615	0.410	0.686	0.665	0.583	0.369
**plot 7**																				
Linear	0.175	0.438	0.528	0.331	0.398	0.061	0.219	0.448	0.255	0.135	0.053	0.002	0.001	0.009	0.004	0.173	0.033	0.002	0.022	0.081
Logarithmic	0.441	0.830	0.845	0.472	0.544	0.093	0.396	0.636	0.361	0.153	0.002	0.000	0.000	0.001	0.000	0.126	0.004	0.000	0.006	0.067
Quadratic	0.323	0.647	0.660	0.407	0.476	0.022	0.325	0.546	0.295	0.112	0.025	0.000	0.000	0.010	0.004	0.336	0.025	0.002	0.034	0.170
Power	0.357	0.789	0.794	0.450	0.503	0.093	0.388	0.591	0.361	0.184	0.007	0.000	0.000	0.002	0.001	0.125	0.004	0.000	0.006	0.049
S	0.388	0.820	0.796	0.358	0.414	0.050	0.346	0.497	0.277	0.099	0.004	0.000	0.000	0.007	0.003	0.194	0.008	0.001	0.017	0.117
Exponential	0.118	0.360	0.410	0.278	0.308	0.060	0.185	0.364	0.227	0.142	0.097	0.006	0.003	0.017	0.012	0.175	0.048	0.006	0.030	0.075

Logistic	0.118	0.360	0.410	0.278	0.308	0.060	0.185	0.364	0.227	0.142	0.097	0.006	0.003	0.017	0.012	0.175	0.048	0.006	0.030	0.075
**plot 8**																				
Linear	0.094	0.204	0.242	0.313	0.320	0.164	0.218	0.384	0.373	0.384	0.151	0.059	0.043	0.022	0.020	0.084	0.053	0.011	0.012	0.011
Logarithmic	0.146	0.491	0.532	0.543	0.546	0.579	0.392	0.605	0.586	0.542	0.098	0.003	0.002	0.002	0.002	0.001	0.010	0.001	0.001	0.002
Quadratic	0.017	0.316	0.321	0.391	0.400	0.421	0.215	0.454	0.447	0.445	0.362	0.050	0.047	0.026	0.024	0.020	0.106	0.014	0.015	0.016
Power	0.089	0.469	0.509	0.399	0.403	0.541	0.392	0.463	0.420	0.408	0.158	0.004	0.003	0.009	0.009	0.002	0.010	0.004	0.007	0.008
S	0.101	0.520	0.594	0.385	0.385	0.660	0.371	0.447	0.389	0.320	0.142	0.002	0.001	0.011	0.011	0.000	0.012	0.005	0.010	0.020
Exponential	0.031	0.167	0.182	0.174	0.178	0.141	0.220	0.214	0.197	0.237	0.256	0.082	0.072	0.077	0.074	0.102	0.052	0.055	0.063	0.044
Logistic	0.031	0.167	0.182	0.174	0.178	0.141	0.220	0.214	0.197	0.237	0.256	0.082	0.072	0.077	0.074	0.102	0.052	0.055	0.063	0.044
**plot 9**																				
Linear	0.008	0.267	0.339	-0.011	-0.046	0.043	-0.061	0.107	0.041	-0.025	0.306	0.020	0.008	0.379	0.593	0.210	0.780	0.108	0.215	0.449
Logarithmic	0.070	0.575	0.729	0.293	0.190	0.376	-0.064	0.280	0.136	0.014	0.159	0.000	0.000	0.015	0.045	0.005	0.857	0.017	0.080	0.285
Quadratic	0.013	0.567	0.730	0.033	0.160	0.294	-0.136	0.199	0.051	-0.071	0.359	0.001	0.000	0.061	0.116	0.034	0.961	0.083	0.272	0.635
Power	0.065	0.567	0.710	0.293	0.193	0.375	-0.065	0.295	0.153	0.035	0.167	0.000	0.000	0.014	0.044	0.005	0.880	0.014	0.067	0.227
S	0.041	0.490	0.646	0.483	0.382	0.518	-0.066	0.282	0.143	0.036	0.214	0.001	0.000	0.001	0.005	0.001	0.955	0.017	0.075	0.225
Exponential	0.005	0.260	0.313	-0.010	-0.044	0.042	-0.062	0.110	0.047	-0.016	0.314	0.021	0.011	0.373	0.579	0.211	0.803	0.105	0.202	0.400
Logistic	0.005	0.260	0.313	-0.010	-0.044	0.042	-0.062	0.110	0.047	-0.016	0.314	0.021	0.011	0.373	0.579	0.211	0.803	0.105	0.202	0.400
**plot 10**																				
Linear	-0.013	0.264	0.230	0.208	0.221	-0.036	-0.005	0.273	0.254	0.225	0.385	0.020	0.030	0.037	0.032	0.518	0.354	0.018	0.023	0.031
Logarithmic	-0.018	0.188	0.161	0.031	0.041	-0.065	0.056	0.116	0.078	0.043	0.412	0.047	0.062	0.238	0.215	0.872	0.184	0.098	0.146	0.209
Quadratic	-0.041	0.267	0.223	0.152	0.166	-0.104	-0.060	0.231	0.202	0.169	0.522	0.045	0.067	0.124	0.110	0.784	0.590	0.062	0.081	0.107
Power	-0.055	0.075	0.037	-0.054	-0.054	-0.066	0.048	-0.025	-0.041	-0.037	0.695	0.150	0.223	0.680	0.672	0.931	0.200	0.450	0.554	0.525
S	-0.066	-0.046	-0.058	-0.047	-0.048	-0.040	0.074	-0.064	-0.057	-0.058	0.910	0.597	0.733	0.605	0.609	0.547	0.151	0.841	0.722	0.737
Exponential	-0.035	0.171	0.135	0.051	0.052	-0.041	-0.012	0.103	0.081	0.099	0.509	0.056	0.081	0.192	0.191	0.555	0.383	0.113	0.141	0.117
Logistic	-0.035	0.171	0.135	0.051	0.052	-0.041	-0.012	0.103	0.081	0.099	0.509	0.056	0.081	0.192	0.191	0.555	0.383	0.113	0.141	0.117
**plot 11**																				
Linear	0.104	0.353	0.384	-0.067	-0.066	-0.047	0.061	-0.061	-0.055	-0.010	0.112	0.007	0.005	0.969	0.944	0.604	0.174	0.790	0.695	0.374
Logarithmic	0.031	0.485	0.570	0.007	-0.005	0.142	-0.067	-0.065	0.016	0.121	0.237	0.001	0.000	0.309	0.355	0.075	0.971	0.888	0.279	0.094
Quadratic	0.078	0.408	0.469	-0.130	-0.129	0.123	0.074	-0.107	-0.130	-0.081	0.222	0.010	0.005	0.924	0.919	0.157	0.228	0.799	0.924	0.677
Power	0.021	0.486	0.533	0.003	-0.010	0.142	-0.066	-0.064	0.014	0.111	0.265	0.002	0.001	0.323	0.375	0.076	0.917	0.858	0.286	0.104
S	-0.062	0.345	0.443	0.186	0.170	0.277	0.007	-0.003	0.168	0.292	0.798	0.008	0.002	0.048	0.056	0.017	0.310	0.344	0.058	0.015
Exponential	0.092	0.308	0.331	-0.067	-0.066	-0.048	0.079	-0.061	-0.057	-0.016	0.126	0.012	0.009	0.998	0.903	0.612	0.145	0.777	0.718	0.402
Logistic	0.092	0.308	0.331	-0.067	-0.066	-0.048	0.079	-0.061	-0.057	-0.016	0.126	0.012	0.009	0.998	0.903	0.612	0.145	0.777	0.718	0.402
**plot 12**																				
Linear	0.073	0.081	0.058	0.112	0.116	-0.038	-0.018	0.096	0.103	0.115	0.153	0.142	0.178	0.103	0.098	0.528	0.411	0.121	0.113	0.100

Logarithmic	0.295	0.443	0.407	0.513	0.519	0.051	0.304	0.494	0.504	0.518	0.014	0.002	0.003	0.001	0.001	0.193	0.013	0.001	0.001	0.001
Quadratic	0.291	0.538	0.512	0.673	0.687	-0.024	0.480	0.699	0.694	0.714	0.017	0.002	0.003	0.000	0.000	0.462	0.004	0.000	0.000	0.000
Power	0.167	0.411	0.417	0.446	0.443	0.007	0.300	0.455	0.452	0.489	0.058	0.003	0.003	0.002	0.002	0.308	0.013	0.002	0.002	0.001
S	0.174	0.453	0.462	0.476	0.471	0.034	0.421	0.483	0.481	0.503	0.054	0.002	0.002	0.001	0.001	0.229	0.003	0.001	0.001	0.001
Exponential	0.025	0.084	0.083	0.106	0.107	-0.048	-0.019	0.106	0.106	0.119	0.253	0.137	0.138	0.109	0.108	0.613	0.416	0.110	0.110	0.096
Logistic	0.025	0.084	0.083	0.106	0.107	-0.048	-0.019	0.106	0.106	0.119	0.253	0.137	0.138	0.109	0.108	0.613	0.416	0.110	0.110	0.096
**plot 13**																				
Linear	0.863	0.417	0.426	0.129	0.126	0.026	0.018	-0.054	-0.040	0.000	0.000	0.003	0.003	0.086	0.089	0.250	0.273	0.678	0.548	0.331
Logarithmic	0.509	0.621	0.707	0.101	0.096	0.089	-0.001	0.024	-0.063	-0.039	0.001	0.000	0.000	0.115	0.121	0.130	0.338	0.212	0.816	0.537
Quadratic	0.878	0.489	0.580	0.080	0.075	0.043	-0.041	-0.057	-0.113	-0.071	0.000	0.004	0.001	0.219	0.227	0.289	0.521	0.577	0.833	0.634
Power	0.521	0.571	0.666	0.093	0.083	0.084	0.004	0.039	-0.064	-0.042	0.001	0.000	0.000	0.125	0.139	0.138	0.318	0.218	0.843	0.654
S	0.226	0.512	0.611	0.009	0.003	0.044	-0.043	0.122	-0.064	-0.065	0.031	0.001	0.000	0.300	0.321	0.207	0.565	0.093	0.850	0.898
Exponential	0.876	0.345	0.363	0.121	0.111	0.026	0.024	-0.053	-0.044	-0.007	0.000	0.008	0.006	0.094	0.104	0.252	0.256	0.666	0.578	0.361
Logistic	0.876	0.345	0.363	0.121	0.111	0.026	0.024	-0.053	-0.044	-0.007	0.000	0.008	0.006	0.094	0.104	0.252	0.256	0.666	0.578	0.361
**plot 14**																				
Linear	0.346	0.523	0.603	0.447	0.462	0.020	0.000	0.532	0.485	0.430	0.010	0.001	0.000	0.003	0.002	0.273	0.333	0.001	0.002	0.003
Logarithmic	0.819	0.868	0.901	0.810	0.816	0.250	-0.019	0.853	0.823	0.785	0.000	0.000	0.000	0.000	0.000	0.028	0.412	0.000	0.000	0.000
Quadratic	0.638	0.773	0.833	0.716	0.726	0.113	-0.073	0.779	0.738	0.686	0.001	0.000	0.000	0.000	0.000	0.181	0.625	0.000	0.000	0.000
Power	0.584	0.742	0.766	0.651	0.656	0.083	-0.027	0.691	0.665	0.696	0.000	0.000	0.000	0.000	0.000	0.146	0.451	0.000	0.000	0.000
S	0.731	0.731	0.738	0.692	0.693	0.146	-0.058	0.708	0.695	0.697	0.000	0.000	0.000	0.000	0.000	0.080	0.684	0.000	0.000	0.000
Exponential	0.161	0.337	0.361	0.249	0.254	-0.030	-0.006	0.287	0.265	0.298	0.069	0.011	0.008	0.028	0.027	0.467	0.358	0.019	0.024	0.017
Logistic	0.161	0.337	0.361	0.249	0.254	-0.030	-0.006	0.287	0.265	0.298	0.069	0.011	0.008	0.028	0.027	0.467	0.358	0.019	0.024	0.017
**plot 15**																				
Linear	0.158	0.485	0.503	-0.038	-0.012	0.223	-0.041	0.148	0.103	0.053	0.064	0.001	0.001	0.528	0.382	0.032	0.533	0.071	0.113	0.189
Logarithmic	0.425	0.778	0.790	-0.062	-0.048	0.100	-0.047	0.648	0.593	0.514	0.003	0.000	0.000	0.802	0.611	0.116	0.600	0.000	0.000	0.001
Quadratic	0.159	0.695	0.675	-0.044	-0.021	0.167	-0.103	0.566	0.537	0.487	0.117	0.000	0.000	0.531	0.455	0.109	0.779	0.001	0.002	0.004
Power	0.432	0.766	0.778	-0.061	-0.045	0.107	-0.042	0.645	0.602	0.522	0.002	0.000	0.000	0.784	0.606	0.109	0.558	0.000	0.000	0.001
S	0.583	0.660	0.694	-0.064	-0.053	-0.015	-0.038	0.813	0.790	0.739	0.000	0.000	0.000	0.838	0.662	0.397	0.529	0.000	0.000	0.000
Exponential	0.156	0.438	0.448	-0.039	-0.018	0.234	-0.040	0.147	0.108	0.059	0.065	0.002	0.002	0.539	0.412	0.028	0.541	0.072	0.107	0.177
Logistic	0.156	0.438	0.448	-0.039	-0.018	0.234	-0.040	0.147	0.108	0.059	0.065	0.002	0.002	0.539	0.412	0.028	0.541	0.072	0.107	0.177
**plot 16**																				
Linear	0.310	0.343	0.387	0.392	0.529	-0.055	0.042	0.367	0.329	0.342	0.012	0.008	0.005	0.004	0.001	0.690	0.211	0.006	0.009	0.008
Logarithmic	0.388	0.763	0.794	0.841	0.886	0.049	0.091	0.842	0.818	0.820	0.004	0.000	0.000	0.000	0.000	0.196	0.127	0.000	0.000	0.000
Quadratic	0.325	0.597	0.611	0.638	0.716	-0.049	-0.013	0.632	0.603	0.595	0.025	0.001	0.001	0.000	0.000	0.550	0.430	0.000	0.001	0.001
Power	0.313	0.678	0.693	0.778	0.824	0.053	0.144	0.762	0.755	0.773	0.011	0.000	0.000	0.000	0.000	0.189	0.074	0.000	0.000	0.000

S	0.254	0.757	0.774	0.859	0.829	0.147	0.157	0.883	0.890	0.892	0.023	0.000	0.000	0.000	0.000	0.072	0.064	0.000	0.000	0.000
Exponential	0.192	0.253	0.267	0.314	0.405	-0.052	0.052	0.275	0.263	0.288	0.044	0.023	0.020	0.011	0.004	0.650	0.190	0.018	0.020	0.015
Logistic	0.192	0.253	0.267	0.314	0.405	-0.052	0.052	0.275	0.263	0.288	0.044	0.023	0.020	0.011	0.004	0.650	0.190	0.018	0.020	0.015

Supplementary Table S4. Spearman's rank correlation coefficients among the values of the functional and species diversity indices FRic, FEve, FDiv, FDis, RaoQ, FDen, Species richness (SR), Shannon, Simpson and Pielou's evenness for the values of the accumulative plots. FRic is positively highly correlated ($p>0.01$) with RaoQ and FDen and negatively correlated with FEve. FEve is positively highly correlated with FDiv and negatively with FDen, while FDiv is negatively correlated with the all the three compositional diversity indices. FDis is positively correlated with RaoQ, as expected, as well as with FDen. RaoQ and FDen are also positively correlated with the three compositional diversity indices, while these three indices are also positively correlated (see Supplementary Table S3).

	Index	FRic	FEve	FDiv	FDis	RaoQ	FDen	Shannon	Simpson	Pielou's evenness
Spearman's rho correlation coefficient	SR	0.723**	-0.231**	0.031	0.581**	0.618**	0.987**	0.658**	0.464**	0.246**
	FRic		-0.327**	0.114	0.137	0.225*	0.795**	0.168	0.043	-0.148
	FEve			0.420**	-0.016	0.03	-0.254**	-0.127	-0.087	0.080
	FDiv				-0.129	0.015	0.061	-0.321**	-0.419**	-0.459**
	FDis					0.976**	0.487**	0.900**	0.814**	0.744**
	RaoQ						0.536**	0.850**	0.754**	0.669**
	FDen							0.574**	0.383**	0.160
	Shannon								0.946**	0.842**
	Simpson									0.945**

*Correlation significant at 0.05 level (two-tailed).

**Correlation significant at 0.01 level (two-tailed).

Supplementary Table S5. Spearman's rank correlation coefficients among the values of the six first axis of PCoA analysis and the initial values of the 26 traits for each species. *: Correlation is significant at the 0.05 level (2-tailed), **: Correlation significant at the 0.01 level (2-tailed).

*Correlation significant at 0.05 level (two-tailed).

	Traits	PCoA axis 1st	2nd	3rd	4th	5th	6th
Spearman's rho correlation coefficient	Longevity	0.492**	0.526**	-.238*	-0.078	0.249*	0.006
	Max Plant height (mm)	0.134	0.253*	0.202	0.130	-0.145	0.032
	Mean Leaf length (mm)	0.192	0.099	0.603**	0.233*	-0.154	-0.112
	Mean Leaf width (mm)	-0.292**	0.443**	0.303**	0.320**	-0.226*	-0.064
	Mean leaf lenght/width	0.423**	-0.375**	0.318**	-0.039	0.101	-0.061
	Life form	-0.485**	-0.527**	0.247*	0.088	-0.271**	0.001
	Growth form	-0.721**	0.108	-0.113	-0.144	-0.197	0.089
	Leaf surface texture	-0.361**	-0.202	0.166	0.017	-0.171	0.524**
	Canopy structure	0.229*	-0.416**	0.349**	0.182	-0.016	-0.145
	Soil acidity	-0.028	-0.319**	0.350**	-0.480**	0.171	-0.017
	Soil nutrient content	0.236*	-0.112	0.282**	-0.621**	-0.125	-0.090
	Soil humidity	0.239*	-0.131	-0.061	-0.532**	0.018	0.038
	Continentality	-0.458**	0.209*	0.131	0.157	-0.432**	-0.304**
	Soil salt content	0.069	-0.154	0.322**	-0.642**	-0.152	0.085
	Light	-0.311**	0.022	0.457**	-0.061	-0.442**	-0.153
	Temperature	0.272**	0.076	0.304**	-0.482**	0.208*	0.043
	Flowering period start (month)	0.100	0.065	0.192	0.108	0.232*	0.251*
	Flowering period end (month)	0.155	0.280**	0.410**	0.012	0.045	0.356**
	Flowering period length	-0.089	0.410**	0.411**	0.120	0.255*	0.417**
	Seed production	-0.080	0.380**	0.190	-0.420**	-0.074	-0.277**
	Seed weight (g)	0.109	-0.109	0.117	-0.017	-0.152	0.217*
	Seed weight (g)	-0.690**	0.377**	-0.182	-0.050	0.188	-0.147
	Flower size	-0.216*	-0.336**	0.241*	-0.060	0.330**	-0.098
	Flower sex	-0.621**	0.508**	-0.091	-0.005	0.143	-0.065
	Pollination type	-0.069	-0.620**	-0.334**	0.162	0.048	-0.173
	Dispersal mode	0.057	-0.313**	-0.060	0.189	-0.239*	0.316**

**Correlation significant at 0.01 level (two-tailed).

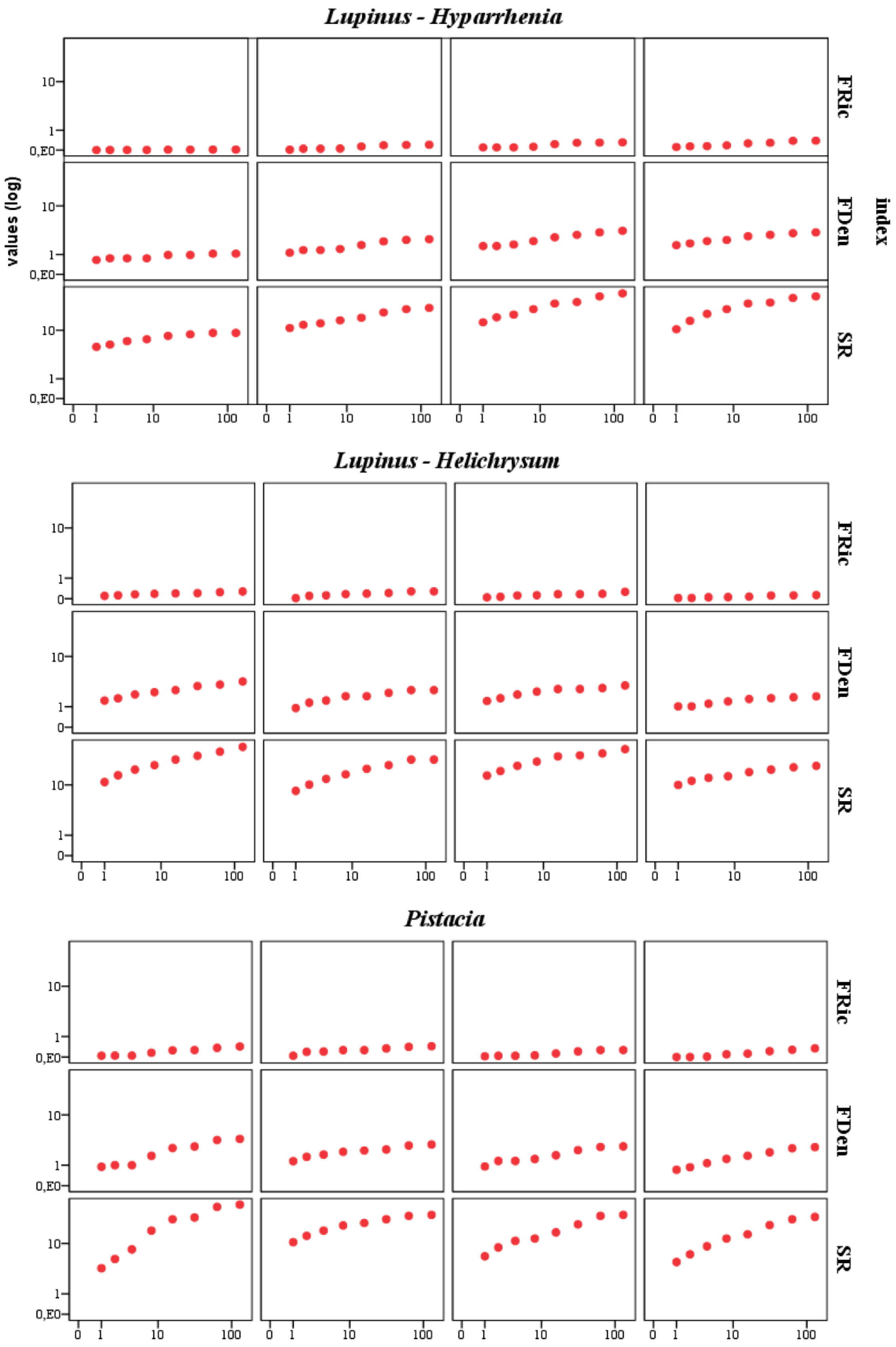

Lupinus - Hyparrhenia
Lupinus - Helichrysum
Pistacia
values (log)
index
FRic
FDen
SR
0,E0
0
1
10
100

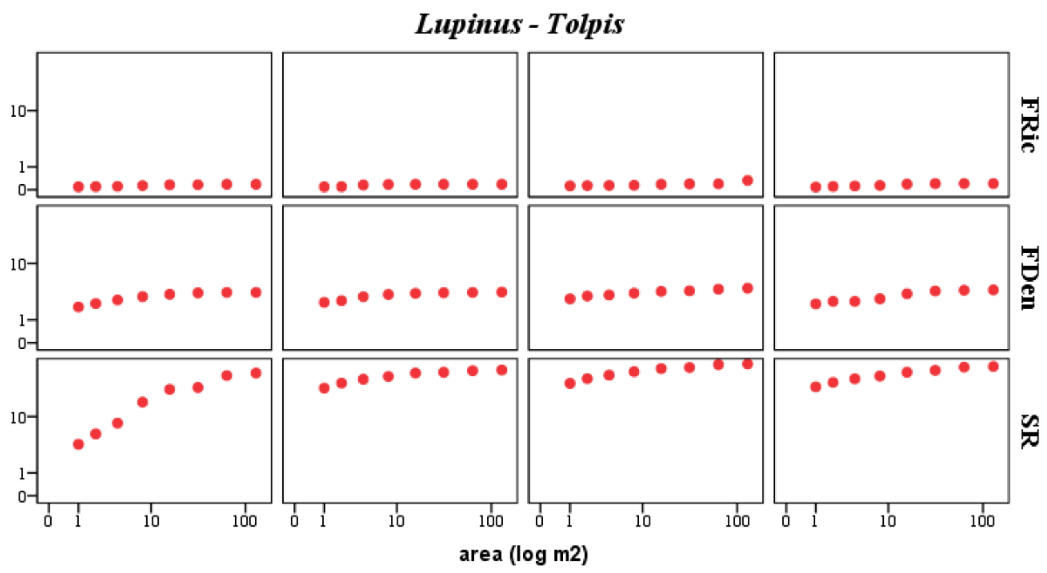

Supplementary Figure S1. The relationship between functional diversity indices values FRic, FDen and area for the four plant communities (*Lupinus angustifolius - Hyparrhenia hirta*, *Lupinus angustifolus - Helichrysum italicum*, *Pistacia lentiscus*, *Lupinus angustifolius - Tolpis barbata*), where each data point represents an accumulative plot (FDACs). The plots' axes are represented in a log-log scale.

Lupinus - Hyparrhenia

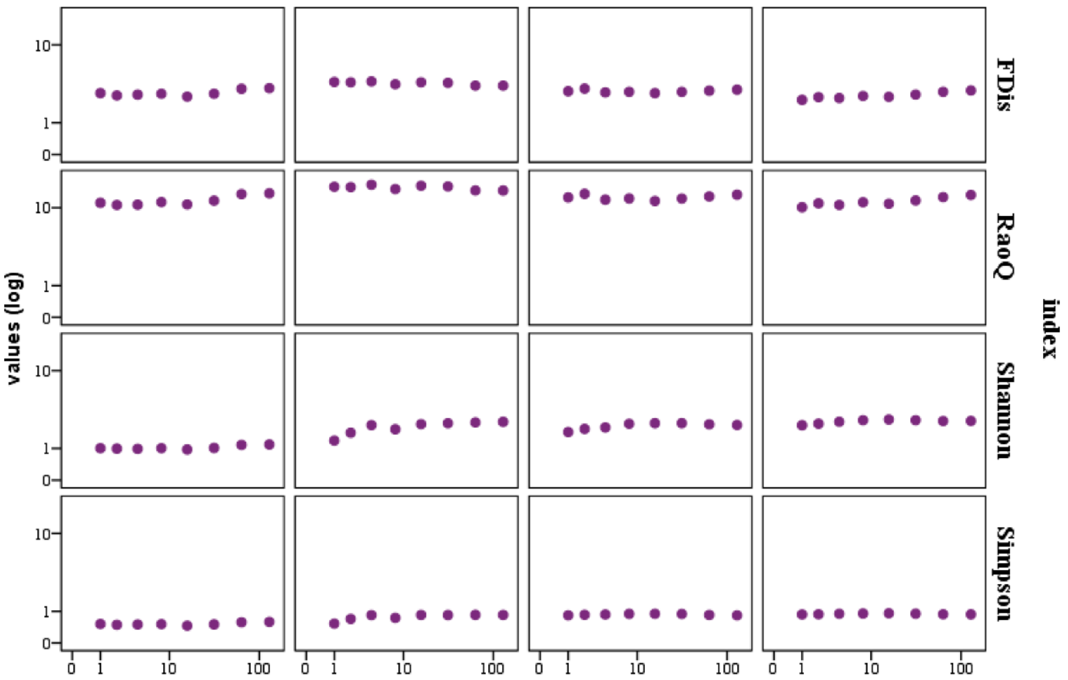

Lupinus - Helichrysum

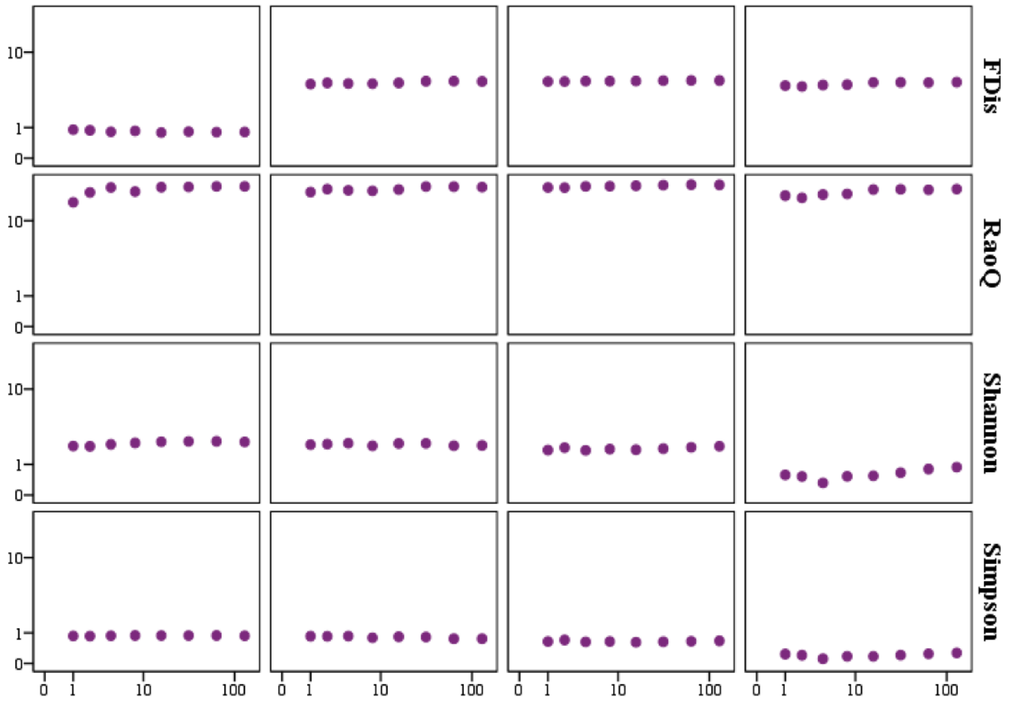

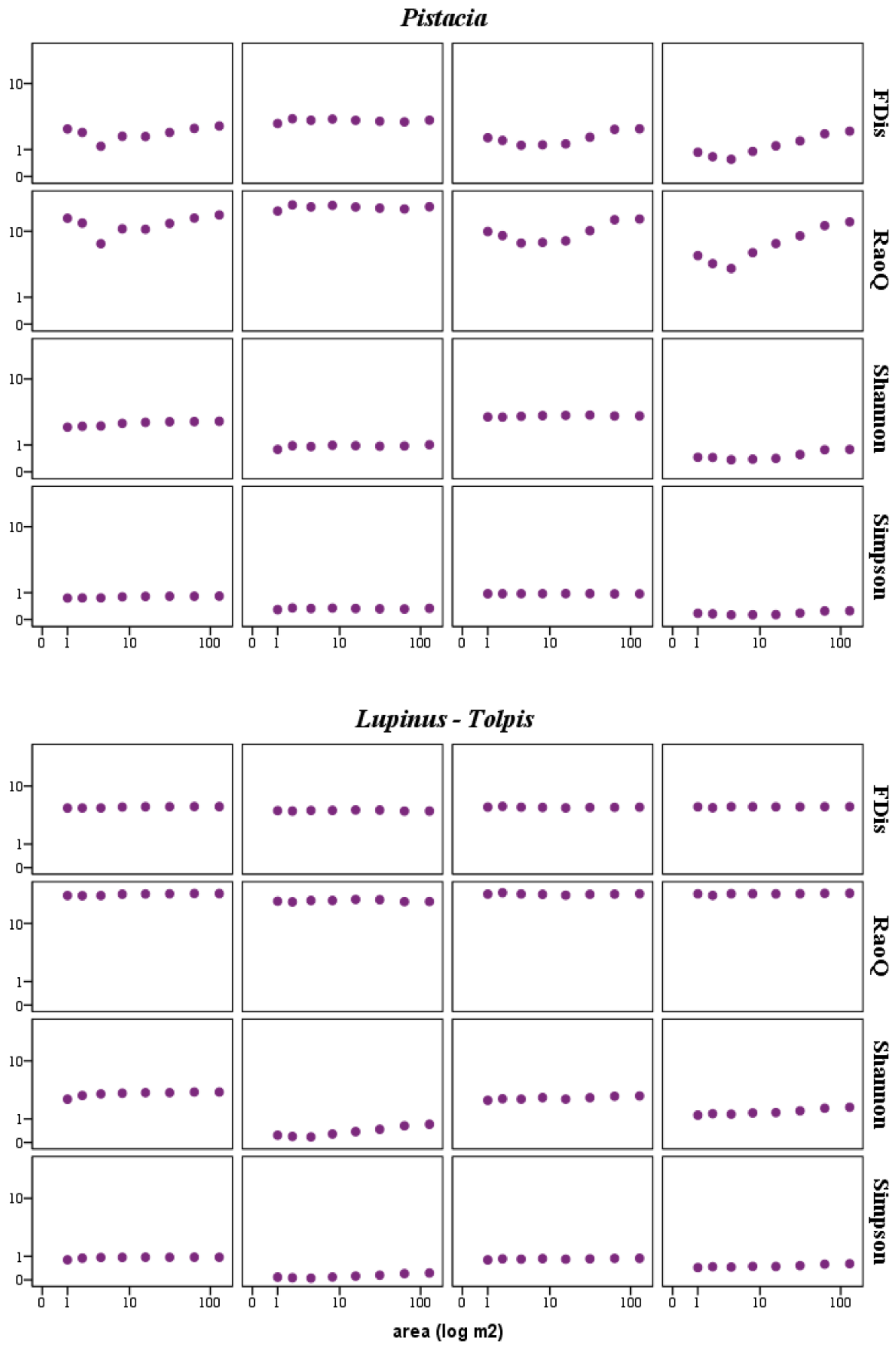

Supplementary Figure S2. The relationship between functional diversity indices values FDis, RaoQ and area for the four plant communities (*Lupinus angustifolius - Hyparrhenia hirta, Lupinus angustifolus - Helichrysum italicum, Pistacia lentiscus,*

Lupinus angustifolius - Tolpis barbata), where each data point represents an accumulative plot (FDACs). The plots' axes are represented in a log-log scale.

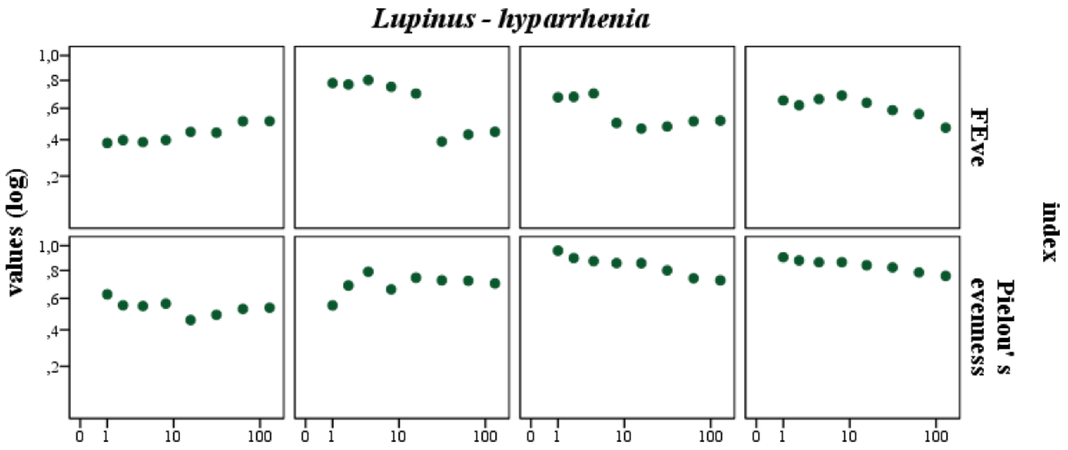

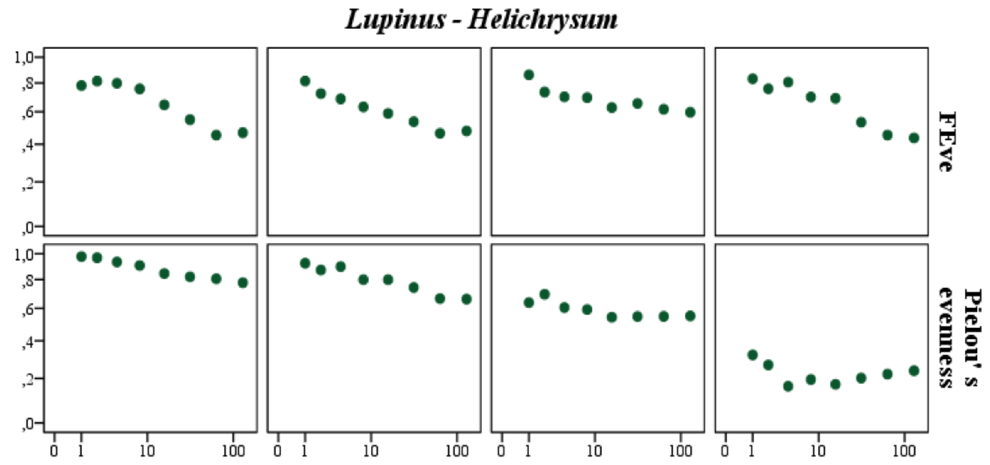

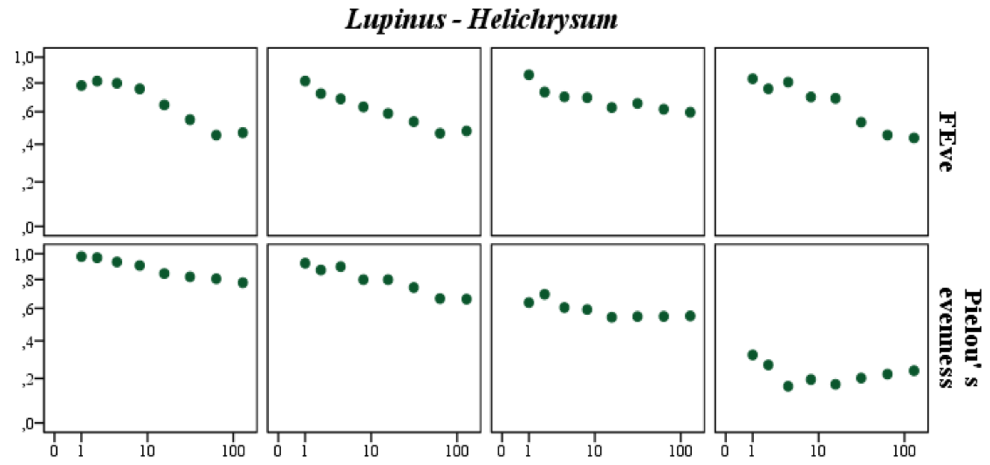

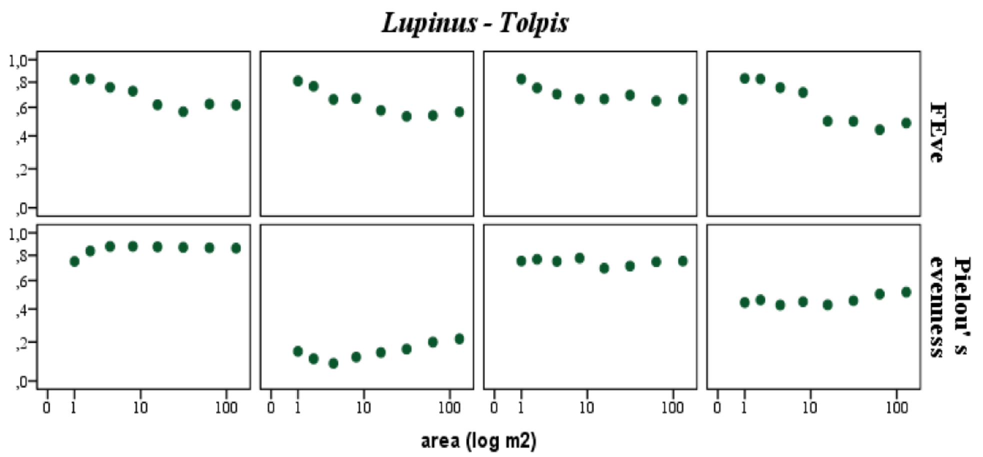

Supplementary Figure S3. The relationship between functional diversity indices values FEve, FDis and area for the four plant communities (*Lupinus angustifolius* - *Hyparrhenia hirta*, *Lupinus angustifolus* - *Helichrysum italicum*, *Pistacia lentiscus*, *Lupinus angustifolius* - *Tolpis barbata*), where each data point represents an accumulative plot (FDACs). The plots' axes are represented in a log-log scale.

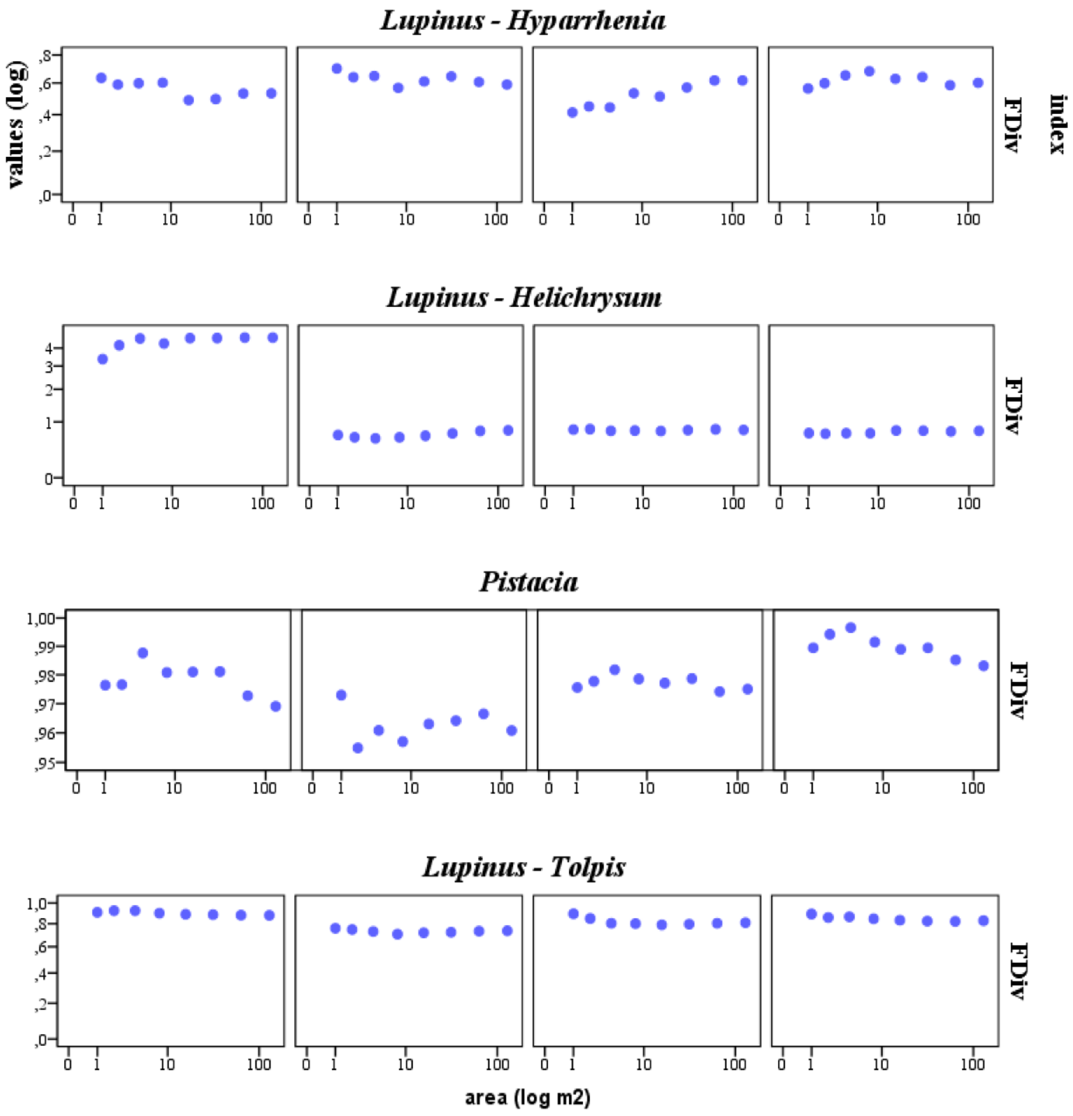

Supplementary Figure S4. The relationship between functional diversity index values FDiv and area for the four plant communities (*Lupinus angustifolius* - *Hyparrhenia hirta*, *Lupinus angustifolus* - *Helichrysum italicum*, *Pistacia lentiscus*, *Lupinus angustifolius* - *Tolpis barbata*), where each data point represents an accumulative plot (FDACs). The plots' axes are represented in a log-log scale.

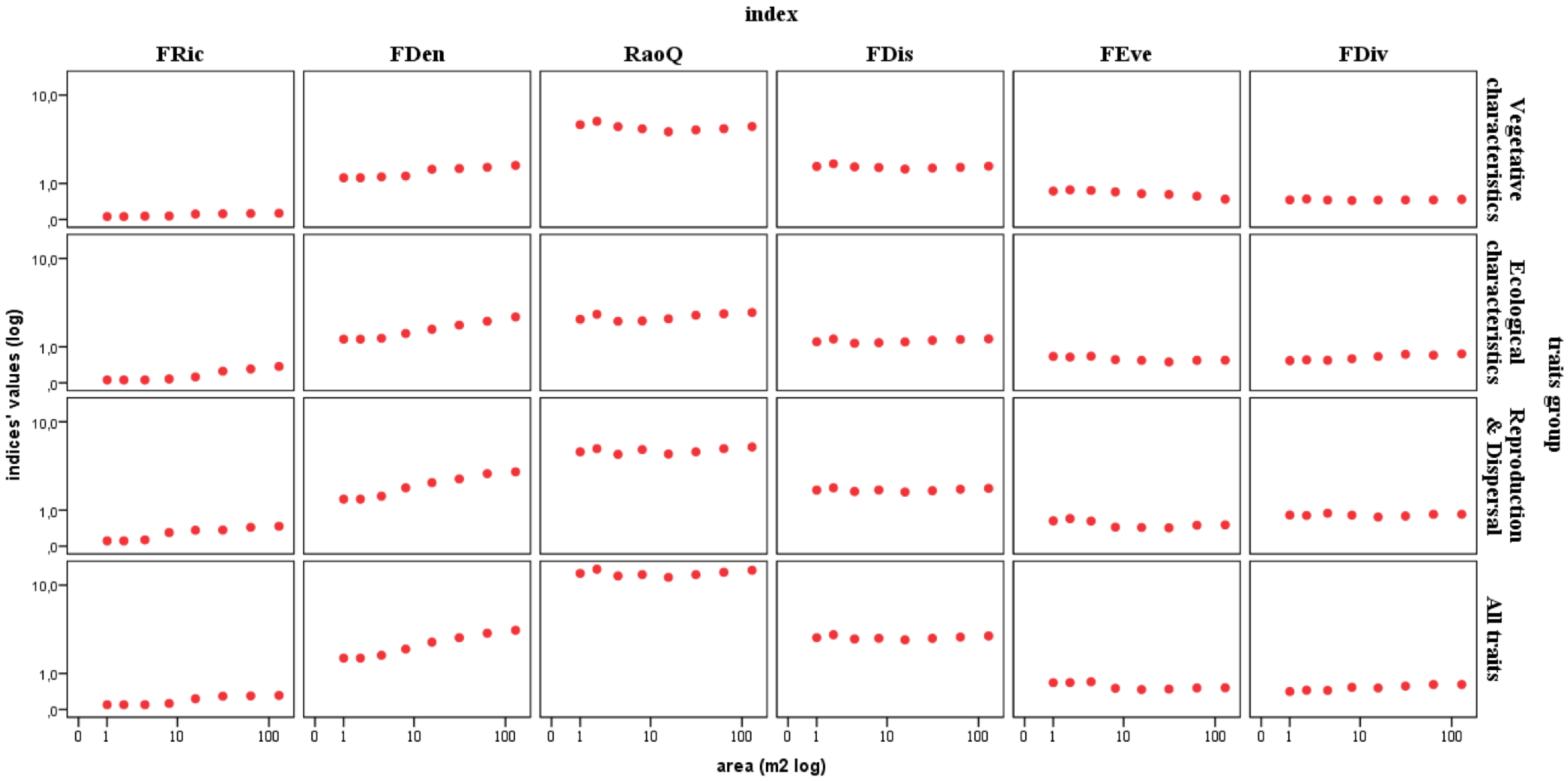
index
FRic
FDen
RaoQ
FDis
FEve
FDiv
Vegetative characteristics
Ecological characteristics
Reproduction & Dispersal
All traits
traits group
indices' values (log)
10,0
1,0
,0
0
1
10
100
area (m2 log)

Supplementary Figure S5. The trait – based functional diversity – area relationship for the six functional diversity indices used in our analysis. In order to investigate the effect of different types and number of traits that can be used in calculating functional diversity, we used four different traits data sets to calculate the FD indices. The first group consisted of the vegetative characteristics, the second of the ecological characteristics, the third group consisted of the reproduction and dispersal characteristics and the last one included all functional traits. We found no significant differentiation in the FDAR pattern, with the exception of the FDiv index that displayed some variation in small scales. The plots' axes are represented in a log-log scale.
